# Pathological Mechanism‐Inspired Biomimetic Nano‐Senotherapy for Reversing Experimental Atherosclerosis in ApoE^−/−^ Mice

**DOI:** 10.1002/advs.75850

**Published:** 2026-05-25

**Authors:** Yuhan Tian, Yanrui Yang, Qiuyu Li, Ying Hu, Qin Hu, Shasha Ran, Siyu Zhou, Xiujuan Yang, Shanshan Li, Qixiong Zhang

**Affiliations:** ^1^ College of Pharmacy and Food Key Laboratory of Research and Application of Ethnic Medicine Processing and Preparation on the Qinghai‐Tibet Plateau Southwest Minzu University Chengdu China; ^2^ Department of Pharmacy Personalized Drug Research and Therapy Key Laboratory of Sichuan Province Sichuan Academy of Medical Sciences & Sichuan Provincial People's Hospital School of Medicine University of Electronic Science and Technology of China Chengdu China; ^3^ Department of Pharmacy Sichuan Provincial People's Hospital East Sichuan Hospital & Dazhou First People's Hospital Dazhou China

**Keywords:** atherosclerosis, MPO inhibitor, navitoclax, senolytics, senotherapy

## Abstract

The greatest challenge in atherosclerosis (AS) management lies in achieving lesion reversal, not merely slowing progression. Senescent cell accumulation—driven by continuous generation and apoptotic resistance—perpetuates plaque pathology and obstructs regression. This study addresses the reversal conundrum through a dual‐action strategy: blocking new senescent cell formation while enhancing clearance of existing senescent cells. We developed a ROS‐responsive dimeric prodrug (K_2_A) from the MPO‐inhibitory tripeptide KYC, which co‐assembles with the senolytics Navitoclax into a nano‐Senotherapy (N@K_2_A). Further cloaked with neutrophil membranes from AS mice, the biomimetic N@K_2_A@NEM precisely targets plaques, responds to local ROS, and orchestrates senescent cell formation and removal. This targeted senotherapeutic intervention demonstrates effective reversal of established experimental AS, offering a potential solution to the field's most pressing clinical dilemma.

## Graphical abstract

The biomimetic self‐assembly nanomedicine reversing atherosclerosis via senotherapy strategy.

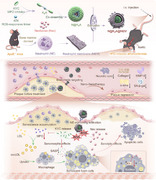

## 1 Introduction

Atherosclerosis (AS) is the most common pathological basis underlying a wide range of cardiovascular diseases (CVDs) [1]. In particular, once the vulnerable plaque falls off, it will directly lead to the occurrence of disabling and fatal CVD such as myocardial infarction and stroke [2, 3, 4]. Statistics show that AS‐related CVDs deaths account for more than 40% of all disease‐related mortality, having already surpassed cancer as the leading cause of death worldwide [3, 5]. Undoubtedly, reversing AS represents one of the most direct and critical strategies for preventing CVD, holding significant research importance. Current treatments for AS include surgical and pharmacological interventions. Surgical approaches involve revascularization procedures such as angioplasty, stenting, or bypass grafting to reopen narrowed or blocked vessels. However, these invasive measures do not address the root cause and are often associated with the need for repeat procedures and poor patient tolerance. Pharmacological therapies mainly consist of lipid‐regulating drugs [6, 7], anti‐inflammatory or anti‐oxidant agents [8], and anti‐platelet drugs [9]. Notably, existing medications do not act directly on AS lesions, which can lead to systemic side effects. Moreover, none of these drugs effectively reverse established plaques, often necessitating long‐term or even lifelong administration—a requirement that in turn exacerbates the risk of drug‐related toxicity.

Precise reversal of AS is therefore a critical approach to addressing the aforementioned challenges, and achieving this goal requires a focused examination of the pathological processes underlying AS. As is well known, AS is a chronic inflammatory disease of the arterial wall, primarily driven by dyslipidemia and dysregulated inflammation [10]. Its development involves lipid infiltration, endothelial injury, inflammatory response, thrombus formation, and other processes. Specifically, activation by excessive oxidized low‐density lipoprotein (ox‐LDL) triggers endothelial cells and smooth muscle cells to release chemokines, which continuously recruit immune cells such as monocytes and neutrophils from the peripheral circulation to sites of endothelial injury. Under the stimulation of inflammatory factors, macrophages migrate into the subendothelial space, take up ox‐LDL, and transform into foam cells. These foam cells further recruit additional inflammatory cells to the lesion site; these cells also engulf ox‐LDL and subsequently become foam cells, accumulating in the lesion area and creating a vicious cycle. This indicates that the continuous generation of foam cells is what drives the initiation and progression of AS.

Moreover, studies have confirmed that the difficulty in reversing AS is also attributed to cellular senescence [11, 12, 13, 14]. *Science* reported that although a large number of foam cells in the plaques of ApoE^−/−^ AS mice cease proliferating, overactivation of anti‐apoptotic pathways such as Bcl‐2 causes them to become “zombie cells”—that is, senescent cells [14]. These senescent cells persistently secrete inflammatory factors, exacerbating AS and making it difficult to reverse. More critically, senescent cells within plaques also secrete matrix metalloproteinases (MMPs), which degrade the plaque envelope and fibrous cap, leading to plaque rupture and triggering severe cardiovascular events [13]. This suggests that “blocking the generation of senescent cells” and “promoting the clearance of senescent cells” represent a potential strategy for reversing AS. Fortunately, in recent years, therapies targeting senescent cells—collectively termed Senotherapy—have gained widespread attention and research interest. These approaches are mainly divided into senolytics (drugs that eliminate senescent cells) and senomorphics (drugs that modulate the senescence‐associated secretory phenotype, SASP).

Within atherosclerotic lesions, senescent cells secrete chemokines that recruit neutrophils (NE) to the local site [15]. The myeloperoxidase (MPO) released by NE, a key enzyme responsible for generating ox‐LDL [16]. This suggests that inhibiting MPO could reduce ox‐LDL production and thereby decrease the formation of senescent foam cells. The tripeptide N‐acetyl lysyltyrosylcysteine amide (KYC) has been reported as a non‐toxic, highly effective, and specific MPO inhibitor [17]. Studies have shown that KYC can alleviate imiquimod‐induced psoriasis in mice [18], treat experimental autoimmune encephalomyelitis (a model of multiple sclerosis) [19], reduce neuronal and neural stem cell damage in a mouse stroke model [20], attenuate vascular oxidative stress in sickle cell disease mice [21], and enhance revascularization in a diabetic hind‐limb ischemia model [22]. Based on this evidence, we hypothesize that KYC may act as a senomorphics by inhibiting MPO activity, thereby reducing MPO‑catalyzed hypochlorous acid production, diminishing ox‐LDL generation, and ultimately blocking the formation of senescent foam cells. On the other hand, the “zombie” state of foam cells represents another major obstacle to reversing AS. Navitoclax (Nav) is recognized as a potent senolytics [23, 24]. It has been shown to clear senescent cells in aged mice, restore hematopoietic stem cell function [24], reverse cognitive decline [25], and treat various senescence‐related diseases in mice, including scleroderma [26], liver cancer [27], diabetes [28], retinopathy [29], and COVID‐19 [30]. Therefore, we propose to harness the senolytic activity of Nav to eliminate senescent cells as a collaborative strategy.

**FIGURE 1:**
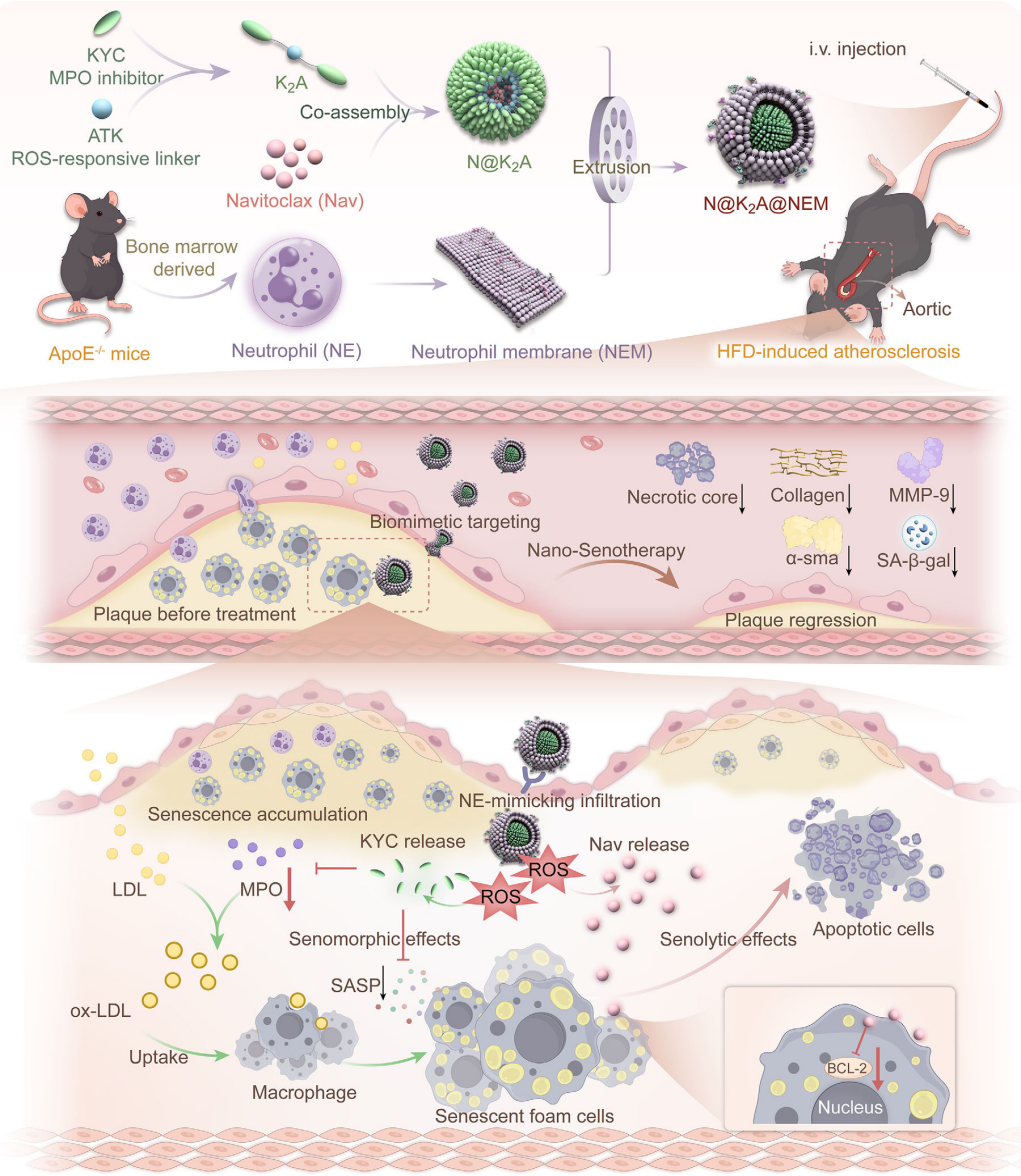
Schematic illustration of the design, biomimetic infiltration, and therapeutic mechanism of N@K_2_A@NEM for atherosclerosis treatment. MPO inhibitor KYC was chemically conjugated to the ROS‐responsive carrier ATK to form the dimeric prodrug K_2_A, which co‐assembled with the senolytic agent Navitoclax (Nav) to generate N@K_2_A nanoparticles. Neutrophil membranes (NEM) derived from bone marrow neutrophils of ApoE^−/−^ mice were subsequently coated onto N@K_2_A via co‐extrusion to obtain N@K_2_A@NEM. After intravenous administration, N@K_2_A@NEM exhibits prolonged circulation time and NE‐mimicking infiltration into inflamed atherosclerotic plaques, mediated by membrane‐associated adhesion and chemotactic proteins. Upon accumulation within the plaque microenvironment, elevated ROS levels trigger the disassembly of ATK, resulting in the on‐site release of Nav and KYC. KYC suppresses MPO‐driven ox‐LDL production, subsequent senescent foam cell generation and SASP secretion, while Nav selectively eliminates senescent foam cells by inhibiting Bcl‐2. The biomimetic nano‐Senotherapy (N@K_2_A@NEM) ultimately reduced the formation of necrotic core, collagen, MMP‐9, and α‐SMA, thereby reversing established atherosclerotic plaques.

Accordingly, a biomimetic targeted nano‐Senotherapy that can specifically regulate senescent cells was developed to reverse AS (Figure 1) [31]. Briefly, using previously reported prodrug‑derivatized linker [32, 33, 34, 35]—the aromatic‑thioketal linker (ATK) —two KYC molecules were covalently linked to ATK to obtain a dimeric prodrug, K_2_A. The ATK moiety in K_2_A provides sufficient hydrophobic interactions, including *π*‐*π* stacking, enabling it to interact with the hydrophobic Nav and form the nano‐Senotherapy (N@K_2_A). Meanwhile, the ROS‐responsive property of ATK that this nano‐Senotherapy release two synergistic drugs on demand in AS lesions. Furthermore, leveraging the natural recruitment of NE to AS sites, the nano‐Senotherapy was coated with neutrophil membrane (NEM) to impart biomimetic properties, yielding N@K_2_A@NEM. Subsequently, its anti‐senescence efficacy, targeting capability, and therapeutic potential against AS were evaluated both in vitro and in vivo.

## 2 Results and Discussions

### 2.1 Synthesis of KYC Dimeric Prodrug (K_2_A)

**FIGURE 2:**
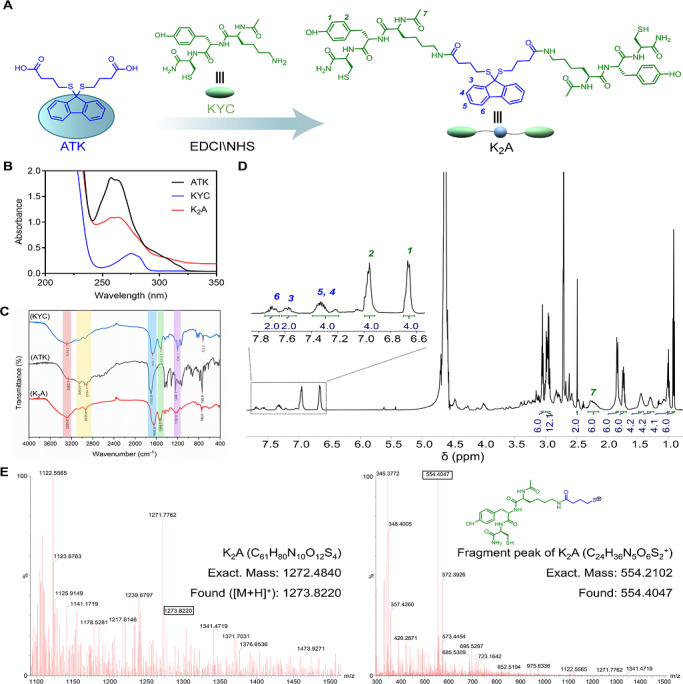
Preparation and characterization of KYC dimeric prodrug (K_2_A). (A), The synthetic route of K_2_A. (B), UV‐vis spectra of reactant (KYC, ATK) and products (K_2_A). (C), FT‐IR spectra of KYC, ATK, and the synthesized K_2_A. (D), ^1^H NMR spectrum of K_2_A. (E), ESI‐MS spectrum of K_2_A and its fragments.

To achieve co‐loading of KYC and Nav, it is necessary to transform hydrophilic KYC into an amphiphilic compound so that it can act as a self‐assembling component. Our laboratory has previously developed a hydrophobic, ROS‐responsive linker, abbreviated as ATK, which can be used to modify various homo‐ or hetero‐dimeric prodrugs [32, 33, 34, 35]. Therefore, a simple EDC/NHS‐mediated amidation reaction was employed to prepare the target compound K_2_A (Figure 2A).

To confirm the chemical structure of the obtained product K_2_A, multiple characterization techniques were employed. Figure 2B shows the UV‐vis spectra of ATK, KYC, and K_2_A. Compared with KYC, K_2_A exhibits a maximum absorption peak at 250–275 nm, which aligns with the characteristic absorption of ATK, confirming the presence of the aromatic conjugate ring unique to ATK in the product. Figure 2C displays the FT‐IR spectra of KYC, ATK, and the synthesized K_2_A recorded in the range of 400–4000 cm^−1^. Compared with ATK and KYC, K_2_A exhibited a newly emerged amide I band at ∼1645 cm^−1^ and an enhanced amide II band in the range of 1500–1600 cm^−1^, indicating the formation of amide linkages between ATK and KYC. Meanwhile, the characteristic C═O stretching vibration of ATK around 1700 cm^−1^ showed a noticeable shift, suggesting the consumption of carboxyl groups during the coupling reaction. In addition, the broad absorption band at ∼3285 cm^−1^ corresponding to N─H stretching further supported successful conjugation. Meanwhile, Figure 2D presents the ^1^H NMR spectrum of K_2_A in D_2_O. Based on chemical shifts and integration ratios, signals were assigned not only to the aromatic and methylene protons from the ATK moiety but also to the aromatic protons and unique methyl protons from the KYC units. Moreover, integration of specific hydrogen atoms from KYC and ATK confirmed a precise 2:1 molar ratio between the two components in the product, hence its designation as K_2_A. Additionally, ESI‐MS spectrometry (Figure 2E) revealed the molecular ion peak of K_2_A (C_61_H_80_N_10_O_12_S_4_, M_w_: 1272.4840, found: [M+H]^+^ 1273.8220) and a fragment peak (C_24_H_36_N_5_O_6_S_2_^+^, M_w_: 554.2102, M^+^ found: 554.4047). These results collectively demonstrate the successful synthesis of KYC dimeric prodrug K_2_A.

Furthermore, to verify whether K_2_A retains the ROS responsiveness of ATK, an adequate amount of K_2_A was subjected to complete hydrolysis in H_2_O_2_, and the hydrolysis products were analyzed by mass spectrometry. The ESI‐MS data (Figure S1A) of the hydrolyzed K_2_A showed molecular ion peaks corresponding to γ‐thiobutyrolactone (M_w_: 102.1544; found: [M+H]^+^ 103.2423), 9‐fluorenone (M_w_: 180.1510; found: [M+H]^+^ 181.0981), and the prototype drug KYC (M_w_: 453.2046; found: [M+H]^+^ 454.0663). Therefore, it can be inferred that the ROS‐responsive hydrolysis process of K_2_A proceeds as shown in Figure S1B. Upon exposure to ROS, the ATK linker is cleaved. Subsequently, the thiolated KYC intermediate undergoes an intramolecular thioester reaction, releasing a molecule of γ‐thiobutyrolactone and regenerating the active KYC.

These results collectively demonstrate that the prodrug K_2_A was successfully synthesized and chemically characterized, and that it effectively releases the active drug KYC under ROS stimulation, thereby laying a solid foundation for subsequent design.

### 2.2 Molecular Dynamics Simulation Study of the Self‐Assembly Process of K_2_A and Nav

**FIGURE 3:**
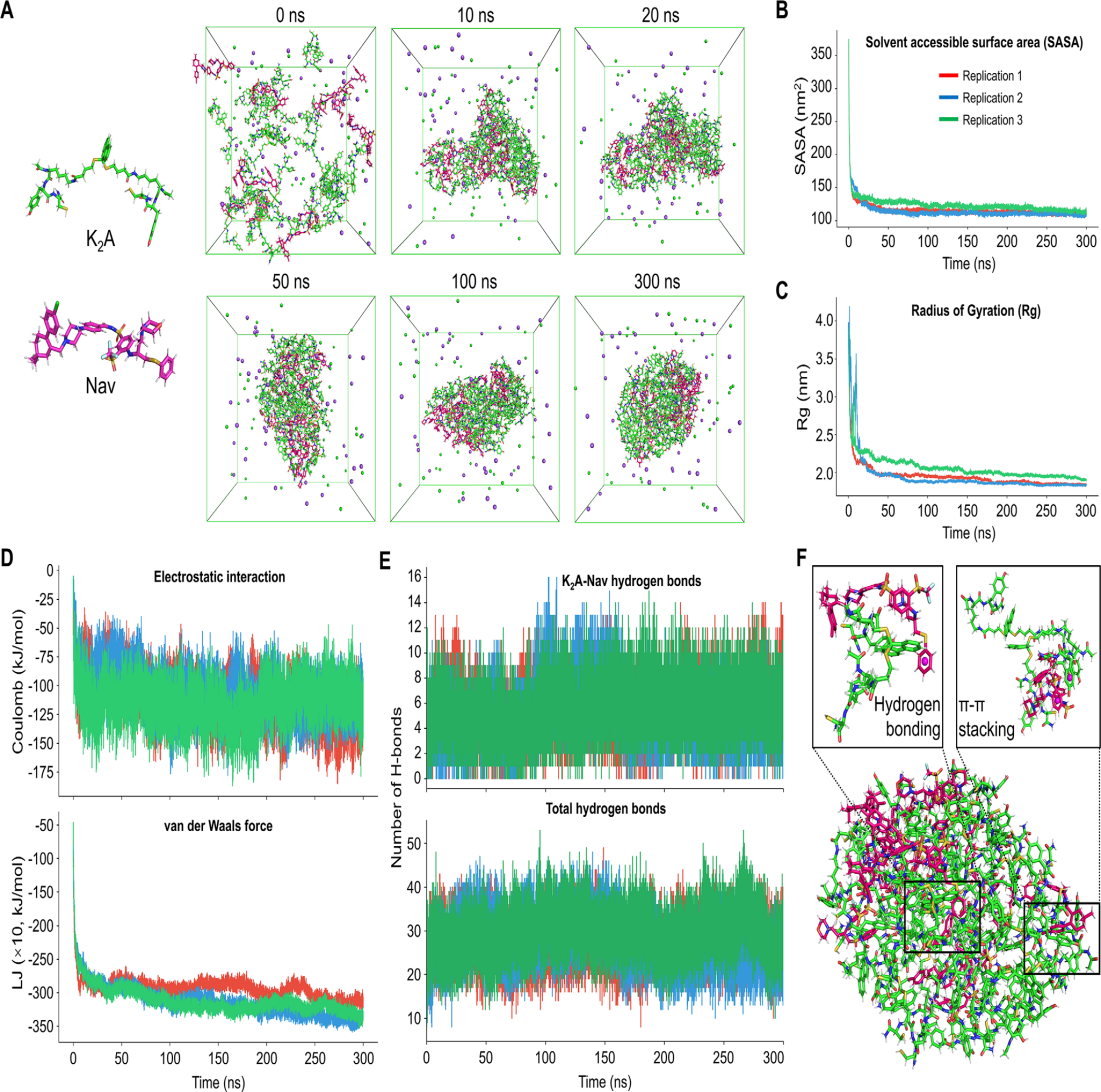
Molecular dynamics simulations and analysis of the self‐assembled system composed of K_2_A and Nav. (A), MD simulations illustrating the self‐assembly process of K_2_A (green) and Nav (red) from 0 to 300 ns. (B–E), Changes in Solvent accessible surface area (B), radius of gyration (C), non‐bonding interaction energy (D), and hydrogen bond (E) in molecular dynamics simulations (F), Predicted interaction modes between K_2_A and Nav, including *π*–*π* interactions and hydrogen bonding.

To predict whether K_2_A and Nav could self‐assemble, all‐atom molecular dynamics (MD) simulations were performed. The self‐assembly process and the interactions between the two components were investigated. Initially, K_2_A and Nav molecules were randomly packed in a cubic box with a side length of 90Å (Figure 3A). A snapshot of an aggregate was selected from the simulation trajectory to illustrate the stacking mode of K_2_A and Nav in the assembled state. In the initial state, all molecules were randomly dissociated.

The simulations demonstrate spontaneous co‑assembly of K_2_A and Nav into a compact nanostructure. Across three independent runs (Figure S2), the solvent‑accessible surface area (SASA) exhibits a consistent two‑stage decline (Figure 3B). Initially (0–20 ns), SASA drops steeply from ∼370 to ∼140 nm^2^, followed by a gradual contraction (50–300 ns) to a stable plateau of 110–120 nm^2^. This marked surface reduction is a classic signature of hydrophobically driven aggregation, as exposed hydrophobic backbones and aromatic rings become sequestered within the assembly core. Evolution of the radius of gyration (Rg) further reflects densification (Figure 3C). Rg values plunge from ∼4.0 nm to below 2.5 nm within the first 30 ns and subsequently exhibit a slow, subtle decrease, converging to 1.8–2.0 nm. This trajectory confirms formation of a tightly packed, stable entity rather than a loose transient cluster. Non‑bonded interaction energies (Figure 3D) reveal two‑stage kinetics. During rapid aggregation (0–50 ns), LJ van der Waals energy plummets from −500 to −2800 kJ/mol, underscoring hydrophobic collapse. In the subsequent densification phase (50–300 ns), LJ energy drifts further downward to −3000 to −3500 kJ/mol, indicating internal structural optimization. In contrast, Coulombic electrostatic energy remains consistently low (−50 to −150 kJ/mol) with no temporal trend, confirming that hydrophobic and van der Waals forces dominate assembly, while electrostatics provide only marginal stabilization. Hydrogen‑bond analysis (Figure 3E) details drug‑loading interactions. K_2_A–Nav hydrogen bonds form rapidly and persist with high‑frequency fluctuations (2–12 bonds) throughout 300 ns, acting as directional anchors that secure Nav within the hydrophobic core. Concurrently, total system hydrogen bonds escalate early and remain abundant (15–45 bonds), creating a dense internal scaffold. This network cooperates with hydrophobic forces to exclude water and confer mechanical rigidity and long‑term stability. Figure 3F reflects typical examples of interaction forces in the N@K_2_A structure: hydrogen bonding and *π*–*π* stacking.

At pH 5.0 (inflammatory microenvironment), self‑assembly still occurs (Figure S3A,B), but the driving forces are remodeled (Figure S3C–G). In Figure S3C, while van der Waals energy remains substantial (∼ −1879 kJ/mol), it is markedly weaker than at neutral pH (∼−3000 kJ/mol). Meanwhile, Coulombic energy intensifies nearly threefold (from −110 to −320 kJ/mol), as Nav likely becomes a zwitterion under acidic conditions. This charge introduction mandates electrostatic matching, partially sacrificing optimal hydrophobic packing and *π*–*π* stacking. Consequently, the assembly adopts a looser morphology: Rg stabilizes at ∼2.25 nm (vs. 1.8–2.0 nm at neutral pH), reflecting electrostatic repulsion and increased hydration space (Figure S3D). Nonetheless, SASA still declines sharply and stabilizes at 130–140 nm^2^, confirming intact aggregation (Figure S3E). Hydrogen‑bond networks remain robust and dynamic, indicating that the increased polar character provides additional anchoring sites (Figure S3F,G). In summary, pH 5.0 yields a somewhat swollen yet fully assembled state.

### 2.3 Preparation and Characterization of Nano‐Senotherapy (N@K_2_A)

**FIGURE 4:**
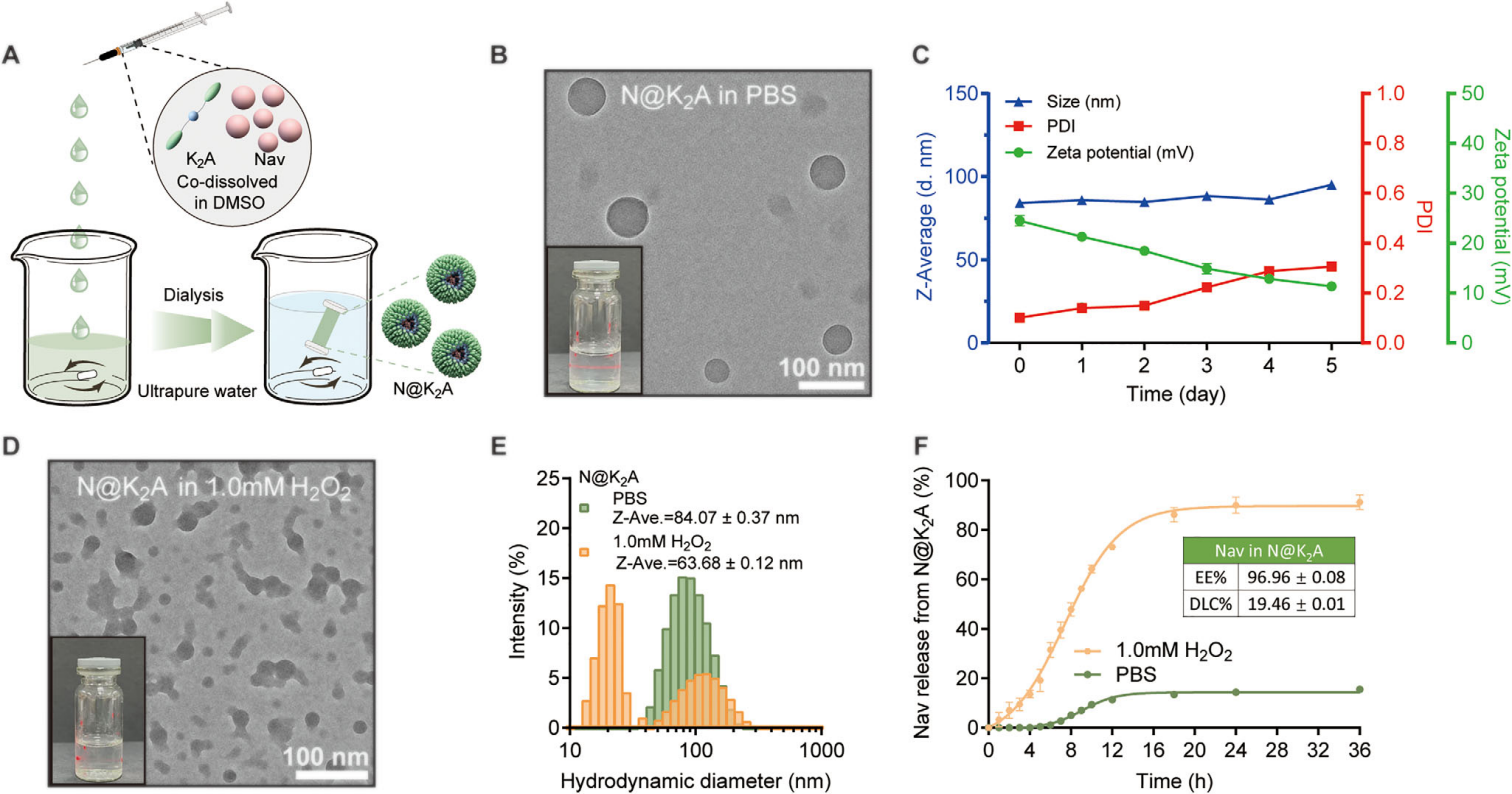
Preparation, characterization, and assembly mechanism of nano‐Senotherapy (N@K_2_A). (A), Schematic illustration of the preparation of N@K_2_A. (B), Representative TEM image and suspension of N@K_2_A in PBS. (C), Time‐dependent changes in Z‐Average, PDI, and zeta potential of N@K_2_A. (D), Representative TEM image and suspension of N@K_2_A in 1 mM H_2_O_2_. (E), Hydrodynamic size distribution of N@K_2_A measured by DLS in PBS or 1 mM H_2_O_2_. (F), ROS‐responsive drug release profile of Nav from N@K_2_A in PBS or 1 mm H_2_O_2_. Scale bar, 100 nm. All data are presented as the mean ± SD (*n* = 3).

Considering the results of MD simulation, the co‐loading of K_2_A and Nav was achieved through precipitation method (Figure 4A). K_2_A and Nav were co‐dissolved in a small amount of DMSO and slowly added to water to form a colloidal system, which was purified by dialysis and lyophilized to obtain the designed nano‐Senotherapy (N@K_2_A). Figure 4B shows that N@K_2_A exhibits the Tyndall effect in PBS, indicating its colloidal nature. TEM images reveal a regular spherical morphology, with particle sizes generally below 100 nm. Concurrently, EDS mapping reveals the distribution form of Nav within N@K_2_A. As shown in Figure S4, the Cl and F elements characteristic of Nav are interspersed throughout the nanoparticles (which exhibit a high sulfur content from K_2_A). Combined with the MD simulation results, these findings collectively confirm that N@K_2_A is a hybrid nano‐assembly.

Figure 4C presents the changes in hydrodynamic diameter (Z‑average), PDI, and zeta potential of N@K_2_A measured by dynamic light scattering (DLS) over a period of five days. While the Z‑average remained stable, the PDI increased slightly, suggesting mild aggregation, which is consistent with the gradual decrease in zeta potential. As expected, after incubation with 1 mm H_2_O_2_ for 24 h, N@K_2_A showed obvious morphological alterations from TEM, and the Tyndall effect disappeared (Figure 4D), indicating the collapse of the colloidal system. Correspondingly, DLS measurements (Figure 4E) showed that the Z‐average of N@K_2_A decreased from 84 nm in PBS to 63 nm in 1 mM H_2_O_2_, accompanied by a marked loss of size uniformity. These results demonstrate that N@K_2_A undergoes H_2_O_2_‐triggered hydrolysis. According to standard curve of Nav (Figure S5), the drug loading content (DLC) and encapsulation efficiency (EE) of Nav in N@K_2_A were determined by HPLC to be 19.46%  ± 0.01% and 96.96% ± 0.08%, respectively (Figure 4F). Furthermore, a dialysis‑based release study was conducted to evaluate the cumulative release of Nav from N@K_2_A over 36 h in different media. In PBS, Nav began to be released slowly after 5 h, likely due to mild aggregation or surface‑adsorbed drug, reaching 15.51% ± 0.27% by day three. In contrast, in 1.0 mm H_2_O_2_, the ROS‑responsive hydrolysis of N@K_2_A led to rapid structural disruption, and Nav was released in a near zero‑order kinetics pattern from the beginning, with cumulative release reaching 91.21% ± 2.37% within 36 h.

These findings confirm that N@K_2_A is capable of achieving ROS‐responsive Nav release. Given that 1 mM H_2_O_2_ is representative of the chemical microenvironment in inflammatory diseases such as AS, this feature provides a stimulus‐triggered mechanism for on‐demand drug delivery at AS lesions.

### 2.4 In Vitro Biological Effects of Nano‐Senotherapy

Given the excellent ROS‐responsive drug release properties of N@K_2_A, we aimed to investigate its biological functions in cells. Atherosclerotic lesions, i.e., plaques, contain a large number of foam cells, which are senescent macrophages resulting from excessive uptake of ox‐LDL. Therefore, RAW264.7 cells were selected as the cellular model for subsequent experiments. First, the cytotoxicity of different concentrations of free Nav and N@K_2_A on normal RAW264.7 cells was determined using the CCK‐8 assay kit (Figure S6). As the concentration of Nav increased, the viability of RAW264.7 cells showed a slight decline. Free Nav only reduced cell viability by about 20% at 4 µM, while N@K_2_A required a concentration of 12–16 µM to achieve a similar effect. This indicates that Nav, as a selective senolytic agent, has a very limited impact on the viability of normal cells with proliferative activity, consistent with previous reports [36, 37]. Therefore, 1 µM was selected as the working concentration for subsequent cell experiments, because it provided a relatively safe exposure level with minimal cytotoxicity in RAW264.7 while avoiding the stronger viability loss observed at higher concentrations. Moreover, by encapsulating Nav, N@K_2_A enhances cellular tolerance to the Nav formulation in non‐ROS‐rich environments, thereby improving its safety profile.

**FIGURE 5:**
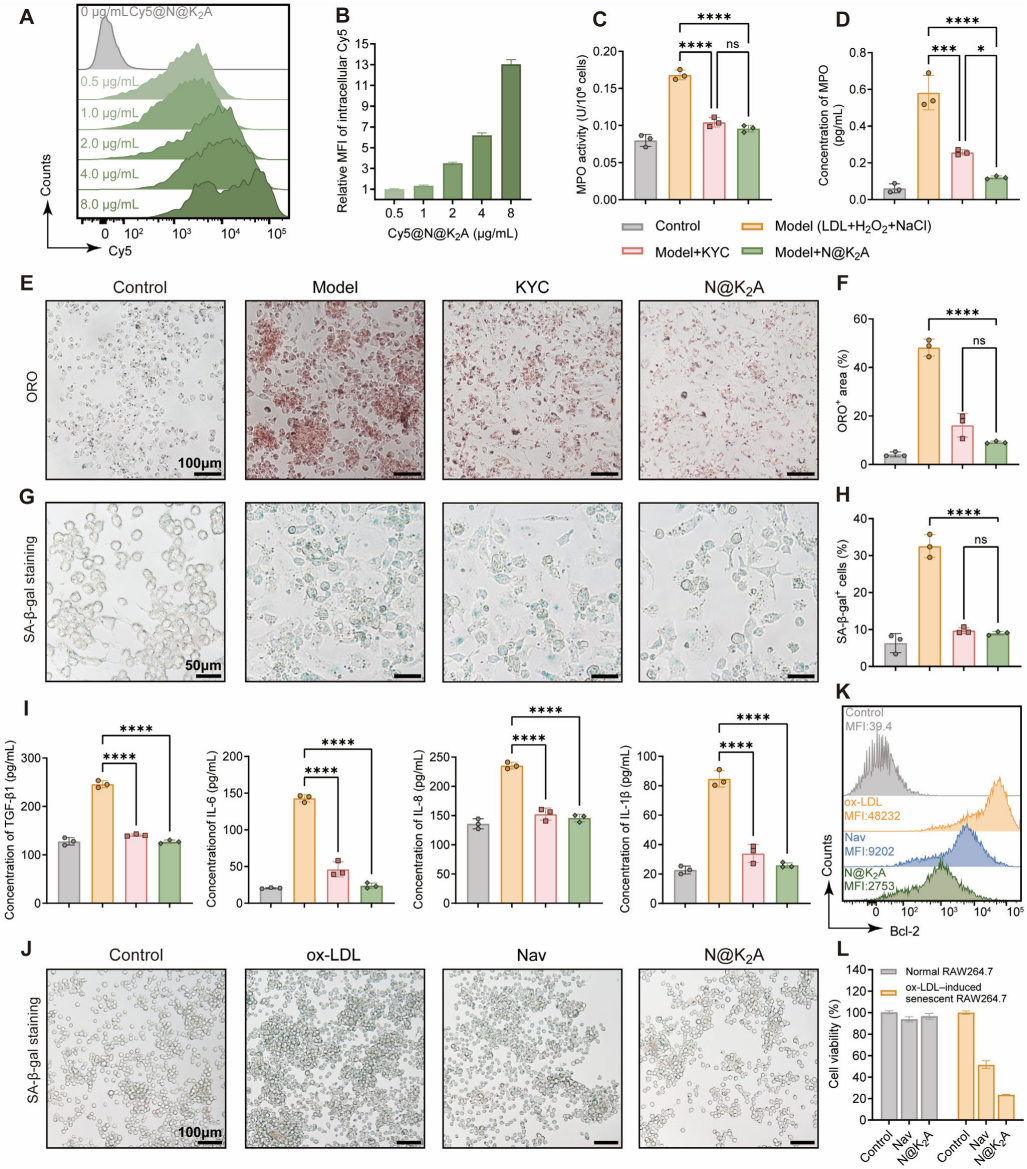
Biological effects of nano‐Senotherapy in senescent cell models in vitro. (A,B), Uptake of different concentrations of Cy5@N@K_2_A in RAW264.7 cells was tested (A) and analyzed (B) by flow cytometry. (C,D), MPO activity (C) and content (D) in RAW264.7 cells (foam cells) undergoing senescence induction by the LDL+H_2_O_2_+NaCl, with or without concurrent different interventions. (E,F), The ORO staining images of senescent RAW264.7 cells with different treatments (E) and statistical analysis of lipid droplets (F). (G,H), The SA‐β‐gal staining images of LDL+H_2_O_2_+NaCl‐induced senescent RAW264.7 cells with concurrent different interventions (G) and statistical analysis of positive cells (H). (I), The expression of SASP in RAW264.7 cells with different treatments. (J), The SA‐β‐gal staining images of ox‐LDL‐induced senescent RAW264.7 cells after treatment with different Nav‐formulations. (K), Following ox‐LDL‐induced senescence in RAW264.7 cells, Bcl‐2 expression after treatment was assessed by flow cytometry. (L), The pro‐apoptotic effect of two Nav‐formulations on ox‐LDL‐induced senescent RAW264.7 cells as determined by CCK‐8 assay, compared with their results in normal RAW264.7 (data from Figure S6). All data are presented the mean ± SD (*n* = 3). For E and J, Scale bar 100 µm, for G, Scale bar 50 µm. ^*^ *p* < 0.05, ^**^ *p* < 0.01, ^***^ *p* < 0.001 and ^****^ *p* < 0.0001.

As a Bcl‐2 inhibitor, Nav must enter cells to exert its effects. Therefore, flow cytometry was used to examine whether N@K_2_A could be internalized by cells. Cy5 was employed as a fluorescent label to prepare Cy5@N@K_2_A. Figure 5A,B show the flow cytometry plots and the corresponding intracellular fluorescence statistics for RAW264.7 cells treated with different concentrations of Cy5@N@K_2_A. As the concentration increased, the mean fluorescence intensity (MFI) of intracellular Cy5 also increased, demonstrating a clear proportional relationship. This suggests that the cellular internalization of N@K_2_A by RAW264.7 cells may occur via free diffusion. Unlike conventional carrier‐dependent nanoparticles, the carrier‐free N@K_2_A likely crosses the cell membrane through simple concentration gradient‐driven transmembrane transport. This mode of uptake is generally independent of carrier protein expression and energy supply, a feature that may offer advantages for intracellular delivery to senescent cells, which often exhibit protein expression variability or compromised energy supply levels.

As per our design rationale: on one hand, KYC as an MPO inhibitor can interfere with MPO‐mediated LDL oxidation (in the presence of H_2_O_2_ and Cl^−^), thereby reducing ox‐LDL generation and directly decreasing the transformation of macrophages into foam cells via ox‐LDL uptake; on the other hand, Nav as a senolytic can downregulate the overexpression of Bcl‐2 in senescent cells, reversing hyper‐activated anti‐apoptotic pathways and restoring the apoptosis process in senescent foam cells. To verify whether N@K_2_A simultaneously retains the biological effects of both KYC and Nav, we designed two experiments: one using LDL+H_2_O_2_+NaCl as a senescence inducer to test whether N@K_2_A can inhibit the conversion of normal RAW264.7 cells into senescent foam cells, and another using ox‐LDL as a senescence inducer to examine whether N@K_2_A can clear already‐formed senescent cells.

Figure 5C,D show the effects of free KYC and N@K_2_A on cellular MPO activity and content. Stimulation with LDL+H_2_O_2_+NaCl upregulated both MPO activity and content in normal RAW264.7 cells, but co‐incubation with either free KYC or N@K_2_A significantly reduced these parameters, confirming that both KYC and N@K_2_A function as intended in inhibiting MPO. Correspondingly, as shown in Figure 5E, Oil Red O (ORO) staining on the treated cells was performed. Control cells displayed no lipid droplets, whereas the model group exhibited obvious lipid accumulation. Figure 5F quantifies the ORO‐positive area (%). LDL+H_2_O_2_+NaCl successfully induced approximately 48.21% ± 2.84% of RAW264.7 cells to take up the ox‐LDL generated by MPO catalysis, while both KYC and N@K_2_A markedly lowered this proportion to 16.18% ± 3.97% and 9.13% ± 0.39%, respectively, with N@K_2_A approaching the control level (4.18% ± 0.81%). Concomitant with the reduction in ox‐LDL uptake, the expression of the senescence marker senescence‐associated β‐galactosidase (SA‐β‐gal) in RAW264.7 cells also declined (Figure 5G,H). Cells were stained using a commercial kit and the percentage of SA‐β‐gal^+^ cells was quantified. Remarkably, KYC or N@K_2_A reduced the percentage of senescent cells from 32.56% ± 2.55% in the model group to 9.74% ± 0.70% and 9.00% ± 0.40%, respectively, restoring it close to the normal group level (6.36% ± 2.12%).

These results demonstrate that N@K_2_A can indeed inhibit MPO, hinder ox‐LDL generation, and consequently reduce the formation of senescent foam cells. Furthermore, as shown in Figure 5I, ELISA data showed that both KYC and N@K_2_A substantially suppressed the upregulation of four SASP‐related cytokines (TGF‐β1, IL‐6, IL‐8, and IL‐1β). Given that a marked reduction in SASP can mitigate the “senescence bystander effect” driven by SASP factors [38], these findings also confirm the excellent ability of N@K_2_A to suppress the generation of senescent foam cells.

In another experiment where ox‐LDL was directly used as a senescence inducer, RAW264.7 cells were successfully induced to become SA‐β‐gal^+^ senescent cells (Figure 5J). Following intervention with Nav or N@K_2_A, the proportion of senescent cells decreased from 31.10% ± 3.77% to 14.66% ± 2.18% and 4.99% ± 1.23%, respectively (Figure S7). Simultaneously, intracellular Bcl‐2 expression levels were measured by flow cytometry (Figure 5K). Notably, normal RAW264.7 cells exhibited very low Bcl‐2 expression, close to baseline (almost negligible), while ox‐LDL stimulation significantly upregulated Bcl‐2. Both Nav and N@K_2_A interventions downregulated Bcl‐2 expression to 19.14% ± 0.56% and 5.72% ± 0.23% of the level in the untreated group, respectively (Figure S8).

Meanwhile, as shown in Figure 5L, approximately 51.55% ± 3.00% and 23.54% ± 0.35% of senescent cells treated with Nav or N@K_2_A still alive. Compared with the survival rates of 93.85% ± 1.94% and 96.67% ± 2.08% observed in normal RAW264.7 cells, this indicates a clear selectivity—Nav and N@K_2_A exhibited significant cytotoxicity only toward ox‐LDL‐induced senescent RAW264.7 cells. This observation aligns with the established consensus that only cells with high Bcl‐2 expression are sensitive to Nav [39, 40].

In summary, the above results demonstrate that the designed N@K_2_A possesses both the ability to inhibit the formation of senescent foam cells and the capacity to promote the selective apoptosis and clearance of existing senescent cells. Thus, N@K_2_A can be regarded as a representative nano‐Senotherapy specifically designed to modulate senescent cells.

### 2.5 Preparation and Characterization of Biomimetic Nano‐Senotherapy (N@K_2_A@NEM)

Given the promising performance of N@K_2_A in in vitro cellular experiments, it is logical that its application effects in the animal level will be considered for evaluation. However, N@K_2_A may lack intrinsic targeting specificity in vivo, which could limit the localized action of Nav and KYC at the lesion site. As shown in the Ly6G immunohistochemical staining results of Figure S9, NE exhibit a significant infiltration behavior in the AS lesions. Therefore, N@K_2_A coated with neutrophil membrane (NEM) was prepared and named N@K_2_A@NEM, in order to achieve the biomimetic targeted delivery of nano‐Senotherapy to the AS lesions in vivo. To this end, NE were first isolated from mouse bone marrow using a density gradient centrifugation‐based cell separation kit (Percoll). Flow cytometry confirmed that CD11b^+^Ly6G^+^ accounted for over 80% of the extracted leukocytes (CD45^+^) (Figure S10A,B). Optical microscopy further identified the isolated cells as NE based on their characteristic polymorphonuclear morphology (Figure S10C). NEM were subsequently obtained using the hypotonic lysis and differential centrifugation method (Figure S10D).

**FIGURE 6:**
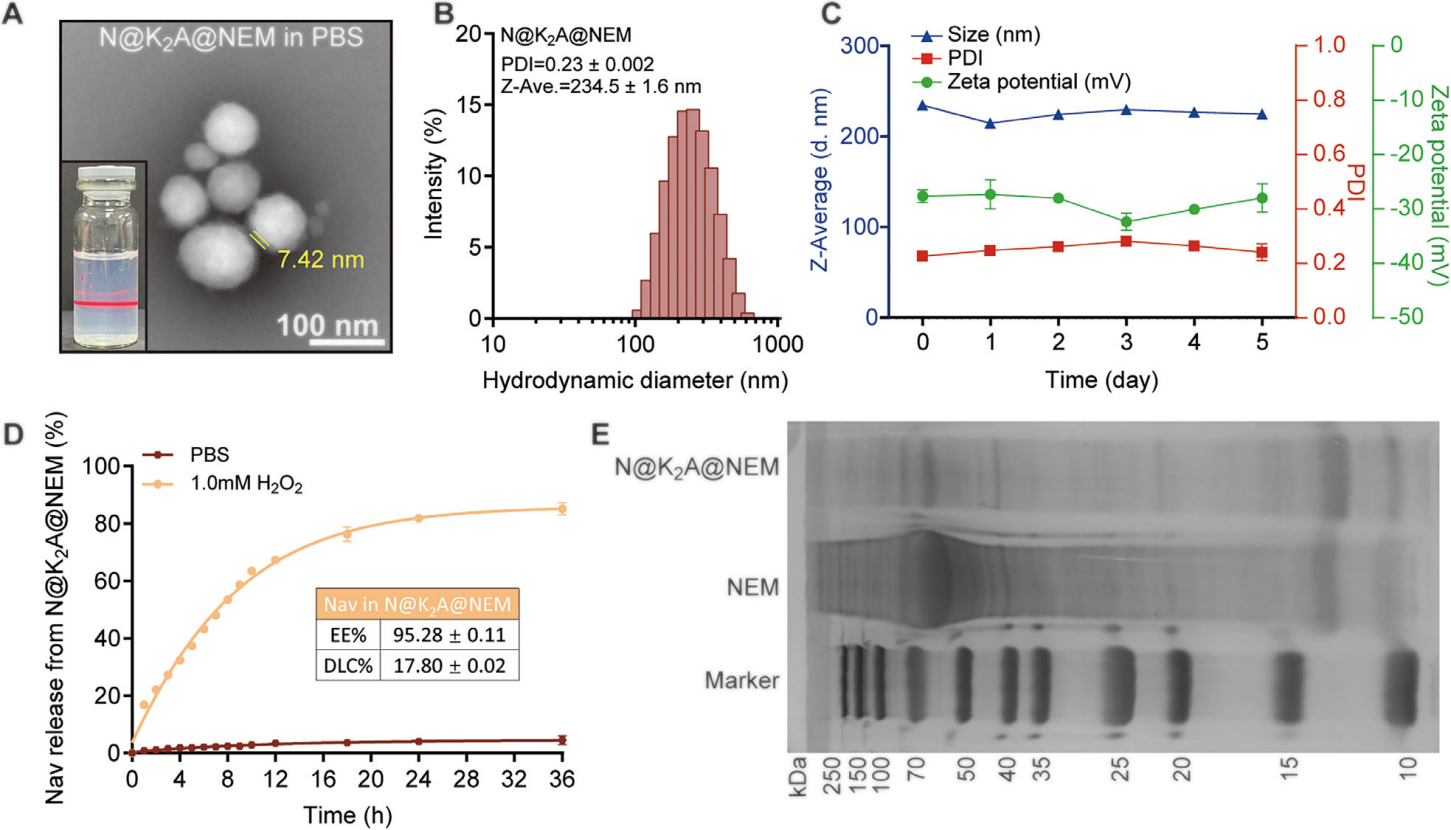
Preparation and characterization of biomimetic nano‐Senotherapy (N@K_2_A@NEM). (A), Representative TEM image and suspension of N@K_2_A@NEM in PBS. (B), Hydrodynamic size distribution of N@K_2_A@NEM measured by DLS in PBS. (C), Time‐dependent changes in Z‐Average, PDI, and zeta potential of N@K_2_A@NEM. (D), ROS‐responsive drug release profile of Nav from N@K_2_A@NEM in PBS or 1 mM H_2_O_2_. (E), SDS‐PAGE image of proteins in NEM and N@K_2_A@NEM. Scale bar, 100 nm. All data are presented the mean ± SD (*n* = 3).

As illustrated in Figure 1A, freshly prepared N@K_2_A and NEM were co‐extruded through a polycarbonate membrane to yield the biomimetic nano‐Senotherapy, N@K_2_A@NEM. The TEM images (Figure 6A,B) revealed spherical particles coated with a layer approximately 7.4 nm thick in PBS, accompanied by a pronounced Tyndall effect and a relatively uniform size distribution (PDI < 0.3). As shown in Figure S11, the Z‐average diameter increased from 84 to 234 nm, and the zeta potential shifted from positive (24.51 ± 0.85 mV) to negative (−27.73 ± 0.82 mV), indicating that NEM coating enhanced the biocompatibility of N@K_2_A. In contrast to N@K_2_A, the particle size, zeta potential, and PDI of N@K_2_A@NEM remained stable without significant fluctuations over a five‐day observation period, demonstrating its superior stability. Furthermore, N@K_2_A@NEM exhibited a higher DLC of 17.80% ± 0.02% and retained the H_2_O_2_‐responsive Nav release profile similar to that of N@K_2_A in the presence of 1 mM H_2_O_2_ (Figure 6D).

The natural migration of NE to AS lesions is attributed to the coordinated action of various chemokine receptors on their surface [41]. To verify that the resulting biomimetic nano‐Senotherapy retains the biological functions of native NE (such as inflammation‐driven chemotaxis mediated by surface proteins), SDS‐PAGE analysis was performed on NEM and N@K_2_A@NEM using a Coomassie Brilliant Blue assay. The results confirmed that N@K_2_A@NEM preserved the surface protein profile of NEM (Figure 6E), providing a material basis for its NE‐mimicking properties.

### 2.6 Selectively Targeting and Accumulation of Biomimetic Nano‐Senotherapy in AS

**FIGURE 7:**
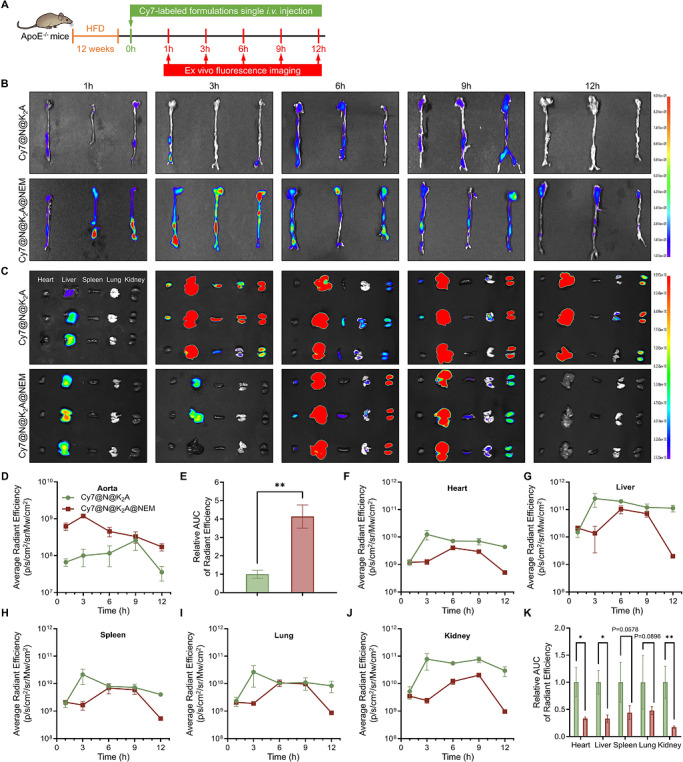
Biodistribution evaluation of nano‐Senotherapy in AS mice. (A), Protocol for the biodistribution experiments. (B), Ex vivo fluorescence photographs of the aorta from AS mice at predetermined time points after administration. (C), Ex vivo fluorescence photographs of the other major organs. (D,E), Statistical analysis of the radiant efficiency changes (D) of Cy7 in aortic tissue and its relative area under the curve (AUC, E) in AS mice treated with different formulations. (F,J), The radiant efficiency changes in heart, liver, spleen, lung, and kidney. (K), Relative AUC of radiant efficiency in each organs. All data are presented the mean ± SD (*n* = 3). ^*^ *p* < 0.05 and ^**^ *p* < 0.01.

Before in vivo application, the hemolytic toxicity of N@K_2_A@NEM was evaluated. As shown in Figure S12, neither nano‐Senotherapy formulation exhibited conspicuous hemolytic toxicity even at a concentration of 200 µg/mL, with N@K_2_A@NEM demonstrating better compatibility. To verify the targeting specificity of the biomimetic nano‐Senotherapy toward AS plaques, a Cy7‐labeled drug was separately prepared for in vivo experiments. Briefly, as shown in Figure 7A, ApoE^−/−^ mice were fed a HFD for 12 weeks and then received a single tail vein injection of either Cy7‐labeled N@K_2_A or N@K_2_A@NEM. Aortas and major organs were collected at predetermined time points (1, 3, 6, 9, and 12 h) for ex vivo fluorescence imaging (Figure 7B,C). As shown in Figure 7D, compared to N@K_2_A, N@K_2_A@NEM exhibited faster, stronger, and more defined accumulation in aortic tissues as early as 1 h post‐injection, gradually declining over time. In contrast, N@K_2_A showed a slower and less stable accumulation pattern, with considerable fluctuation over the observed period. Although its signal intensity became nearly equivalent to that of N@K_2_A@NEM at 9 h, it decreased sharply thereafter. This may be due to the lack of sustained adhesion capability of N@K_2_A, causing it to detach from the aortic wall under shear stress from blood flow. Notably, at every time point examined, the Cy7 average radiant efficiency of N@K_2_A@NEM in aortic tissues was higher than that of N@K_2_A. By integrating the area under the curve (AUC) from 1 to 12 h, the cumulative accumulation of N@K_2_A@NEM in aortic tissue was approximately 4.13‐fold greater than that of N@K_2_A, as summarized in Figure 7E.

Alongside its enhanced performance in aortic tissue, N@K_2_A@NEM also demonstrated reduced accumulation in major organs. As shown in Figure 7F,G, at every time point examined, N@K_2_A@NEM exhibited lower signal intensity in the heart, liver, and kidneys compared to N@K_2_A, indicating less accumulation in these organs. Only in the spleen and lungs did the signal of N@K_2_A@NEM approximate that of N@K_2_A at 6 and 9 h. By 12 h, however, N@K_2_A@NEM showed a clear and rapid decrease across all organs, suggesting that peak concentrations were reached between 9 and 12 h, followed by accelerated clearance. In contrast, N@K_2_A displayed a pharmacokinetic profile characterized by rapid uptake and slow decline, suggesting its clearance from organs may be considerably prolonged. Statistical integration of the AUC for radiant efficiency in major organs from 1 to 12 h revealed that, compared to N@K_2_A, the accumulation of N@K_2_A@NEM in the heart, liver, and kidneys was only 33.23% ± 0.02%, 32.97% ± 0.05%, and 17.28% ± 0.02%, respectively. Although no significant difference was observed in the spleen and lung (p‐value close to 0.05), N@K_2_A@NEM still showed a trend toward lower accumulation. Moreover, as seen in Figure 7H,I, its maximum concentrations in spleen or lung were also lower (only 44.00% ± 0.10% and 47.86% ± 0.06% of N@K_2_A, respectively). These properties are expected to significantly reduce potential off‐target side effects caused by nonspecific distribution.

Therefore, it can be concluded that, owing to the natural recruitment of NE to AS lesions, N@K_2_A@NEM achieves more effective targeting and accumulation within these sites.

### 2.7 Biomimetic Nano‐Senotherapy Combats Aortic Senescence

**FIGURE 8:**
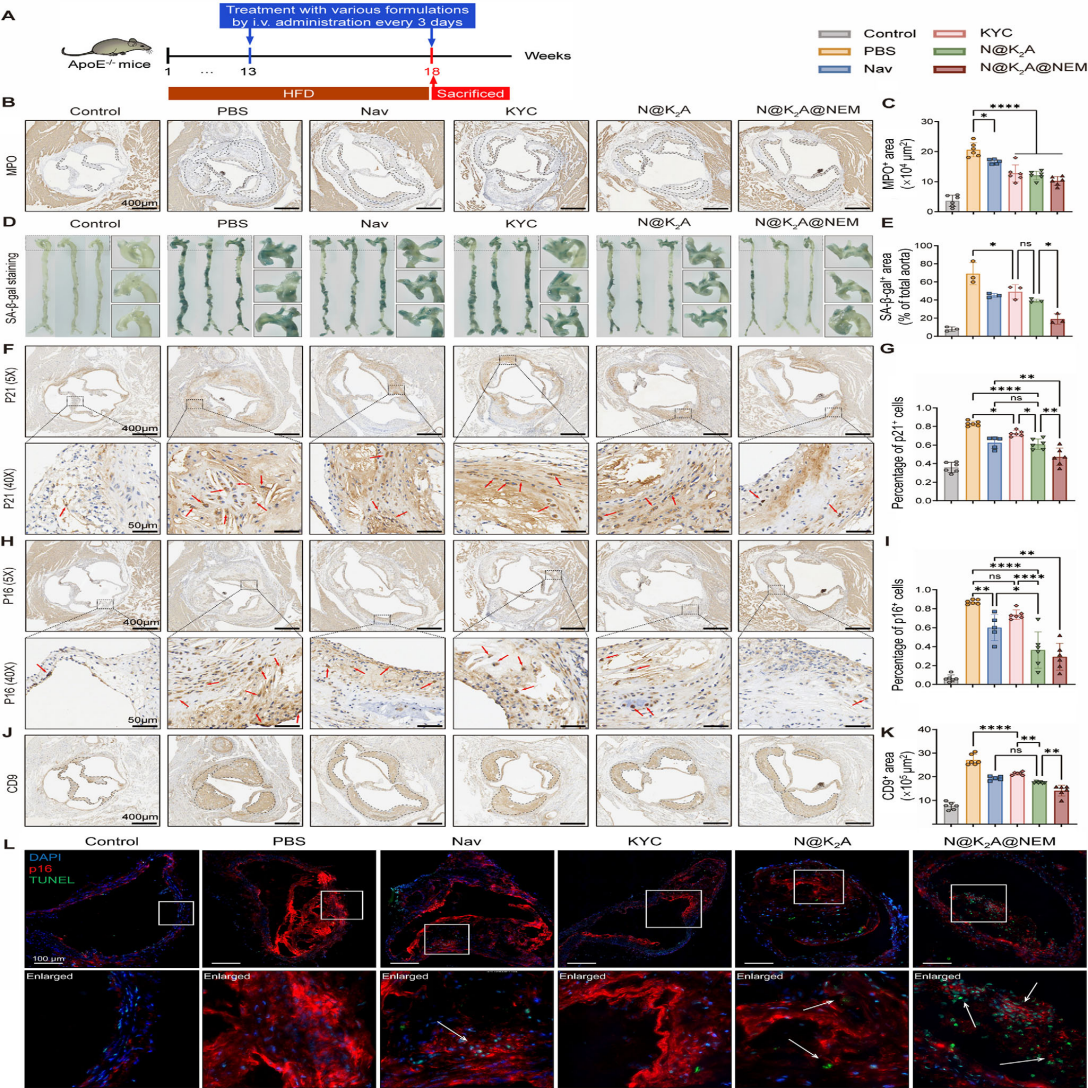
Evaluation of various formulations in relieving senescence of aortic tissue in AS mice. (A), Protocol for these experiments. ApoE^−/−^ mice were fed HFD for 18 weeks. Starting from the 13th week, different formulations were administered via tail vein injection every three days until the end of the 18th week. Euthanize mice and extract aortic tissue, and slice the aortic root tissue. (B,C), Immunohistochemical images (B) and positive area statistics (C) of MPO in the aortic root. (D), Digital photos of SA‐β‐gal staining of the entire aorta tissue and its corresponding aortic arch. (E), Analysis of the relative proportion of SA‐β‐gal^+^ area in entire aorta. (F,G), Immunohistochemical photos of p21 in the aortic root (F) and statistics of p21^+^ cell ratio (G). In the enlarged image, the red arrows represent typical positive cells. (H,I), Immunohistochemical photos of p16 in the aortic root (H) and statistics of p16^+^ cell ratio (I). (J,K), Immunohistochemical images of senescent cell marker CD9 in the aortic root (J) and statistical analysis of its positive expression area (K). (L), Immunofluorescence images showing co‐localization (white arrow) of senescent cells (p16, red) and apoptotic cells (TUNEL, green) in mouse aortic tissues. All data are presented the mean ± SD (*n* = 3, 5, 6). ^*^ *p* < 0.05, ^**^ *p* < 0.01, ^***^ *p* < 0.001, ^****^ *p* < 0.0001, and ns means no significance.

Given the favorable enrichment effect of the biomimetic nano‐Senotherapy in the aorta, its anti‐aortic senescence efficacy in an AS mouse was evaluated. Figure 8A outlines the overall in vivo experimental scheme. ApoE^−/−^ mice were fed a HFD throughout the 18‐week study, during the whole experiment, there was any abnormalities in body weight (Figure S13) and organ index (Figure S14) of AS mice with different treatments. In parallel, we conducted in vivo toxicity assessments for the three Nav‐containing formulations (Figure S15A), with particular attention to the thrombocytopenia that Nav has been reported to induce early in its administration [42]. As shown in Figure S15B, none of the Nav‐containing formulations caused thrombocytopenia. We believe that this is due to our Nav concentration (intravenous injection 0.607 mg/kg) being significantly lower than the reported oral or intraperitoneal administration of 1.5–100 mg/kg [43, 44, 45, 46] in the literature. Similarly, no impairment in liver (ALT and AST) or renal function (CREA and UREA) was observed. In addition, histological examination of liver and kidney tissues revealed no obvious pathological abnormalities, such as inflammation, necrosis, or structural damage (Figure S15C,D). These results collectively indicate that the treatment regimen, including the N@K_2_A@NEM condition, does not induce apparent short‐term systemic toxicity under the tested conditions.

Starting from the 13^th^ week, nano‐Senotherapy was administered via tail‐vein injection every three days until the 18^th^ week, when aortic tissues were collected for subsequent analysis. Figure 8B,C presents representative MPO immunohistochemical images of aortic sections and the corresponding quantitative analysis of positive areas. All treatment groups reduced the MPO‑positive rate in aortic tissues, indicating that Nav can partially clear the MPO^+^ senescent foam cells through its senolytic activity. More importantly, KYC and two KYC‐containing nano‑Senotherapy exhibited stronger suppression of MPO expression. Notably, the N@K_2_A@NEM treatment group showed a dramatic reduction—by nearly 50.32%—compared to the PBS group. More definitive conclusions can be drawn from the ELISA results of MPO and Bcl‑2 shown in Figure S16. Specifically, compared to Nav, N@K_2_A not only retained the Bcl‑2‑inhibitory function of Nav but also, in synergy with the ability of KYC to suppress the emergence of new senescent cells, exerted stronger inhibitory effects on both MPO and Bcl‑2. It is particularly noteworthy that N@K_2_A@NEM produced the greatest suppression for both MPO and Bcl‑2, demonstrating a statistically significant difference compared to N@K_2_A. These findings align with the results presented in Figure 6, indicating that the NEM coating—a biomimetic strategy—potentiates the ability of N@K_2_A@NEM to inhibit MPO and Bcl‑2, thereby providing molecular‑level evidence for its role in blocking the generation of senescent cells and counteracting the anti‑apoptotic state of cells.

Simultaneously, a widely recognized cellular senescence marker, SA‐β‐gal, was detected using a staining kit. Notably, the SA‐β‐gal staining results of aortic tissues in Figure 8D showed significant differences. Compared with the control group, aortas from PBS‐treated AS mice exhibited more extensive and deeper SA‐β‐gal staining, especially in the aortic arch area that is prone to plaque formation. All treatment groups demonstrated varying degrees of reduction in staining intensity. According to Figure 8E, the SA‐β‐gal^+^ area in the PBS group accounted for as high as 69.19%, showing a significant increase over the control group (7.96%), confirming pronounced senescence in the aortas of AS mice. Treatment with Nav and KYC reduced the senescent area. Although the reduction in SA‐β‐gal staining by N@K_2_A was relatively modest, N@K_2_A@NEM produced a more pronounced effect, with a positive area of only 19.13%, representing a decrease of 72.34% compared to the PBS group.

Furthermore, p21 and p16, two other important senescence‐associated markers, were evaluated. Figure  shows representative p21 staining in aortic sections and the proportion of p21^+^ cells. In the magnified images, red arrows indicate p21^+^ nuclei. As quantified in Figure 8G, the proportion of p21^+^ cells in AS mouse aortas increased from 35.90% to 83.14%. While treatment with Nav, KYC, or N@K_2_A each reduced this proportion, no significant difference was observed between N@K_2_A and Nav. In contrast, N@K_2_A showed a clear advantage over KYC. This suggests that the effect of N@K_2_A on p21 is primarily driven by its Nav component, a finding consistent with previous reports [47]. Encouragingly, owing to its enhanced targeting and accumulation in AS lesions, N@K_2_A@NEM yielded an even lower proportion of p21^+^ cells (47.23%), approaching the level seen in control mice (35.90%). Similar conclusions were drawn from the immunohistochemical analysis of p16 (Figure 8H,I): compared to Nav or KYC alone, the nano‐Senotherapy formulations exhibited a clear synergistic effect. Moreover, CD9, reported as another marker overexpressed during AS‐associated senescence, was assessed via immunohistochemistry (Figure 8J‐K). Cytoplasmic regions expressing CD9 were scarce in control mice but significantly increased in PBS‐treated AS mice. Obviously, treatment with N@K_2_A@NEM resulted in the least CD9 expression, indicating the strongest anti‐senescence effect.

More importantly, immunofluorescence imaging of aortic tissue sections revealed that, following intervention with different Nav‐containing formulations, varying degrees of apoptosis were observed among p16^+^ senescent cells within the arterial wall, as indicated by the colocalization of p16 (red) and TUNEL (green) fluorescence signals. In contrast, such colocalization was either absent or exceedingly rare in control mice, PBS‐ or KYC‐treated mice (Figure 8L). These findings demonstrate that Nav‐containing formulation, particularly the biomimetic nano‐Senotherapy, exerts selective pro‐apoptotic effects on senescent cells.

Meanwhile, the SASP serves as a hallmark of senescent cells, directly reflecting the degree of cellular or tissue senescence. On the other hand, SASP can also induce senescence in neighboring normal cells through paracrine signaling. Therefore, evaluating SASP is equally critical. As shown in Figure S17, N@K_2_A@NEM exhibited the most pronounced reduction in fibrosis‐related TGF‐β1 and inflammation‐related IL‐6 and IL‐1β, essentially restoring their levels to those observed in the control group. IL‐8 is a key chemokine that mediates neutrophil recruitment to AS sites and promotes inflammatory responses. Notably, when the neutrophil‐mimetic nano‐Senotherapy accumulated at the lesion sites, it unexpectedly and sharply reduced IL‐8 levels compared to N@K_2_A. This may be attributed to the introduction of exogenous NEM, which likely attenuated tissue‐driven immune cell recruitment, as reflected by the marked decrease in IL‐8.

Collectively, compared to single‐agent treatments, both N@K_2_A and N@K_2_A@NEM demonstrated superior synergistic effects in combating aortic senescence. Leveraging its enhanced targeting specificity and tissue accumulation, N@K_2_A@NEM emerges as a particularly promising candidate for advanced nano‐Senotherapy.

### 2.8 The Effects of Biomimetic Nano‐Senotherapy on Reversing AS

**FIGURE 9:**
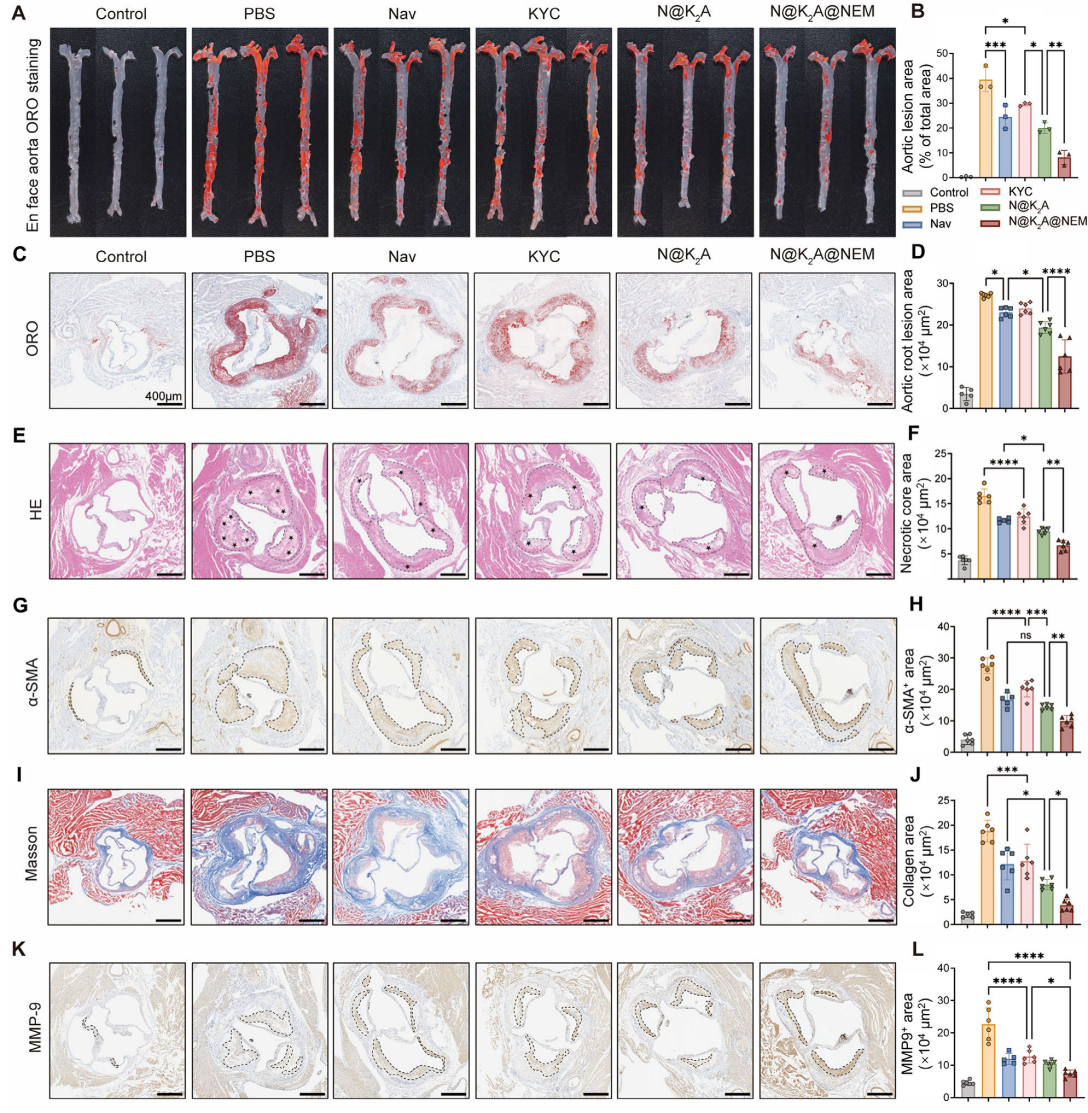
Evaluation of the therapeutic effect of various formulations on HFD‐induced AS mice. (A,B), Photos of aorta of AS mice stained with ORO (A) and relative area statistics of the lesion site (B). (C,D), Images of the aortic root tissue slices stained with ORO (C) and analysis of the lesion area (D). (E,F), H&E staining results of aortic root tissue slices (E) and statistical analysis of necrotic core area (F). (G,H), Immunohistochemical photos of α‐SMA in aortic root tissue (G) and statistical analysis of positive site area (H). (I,J), Masson staining photos (I) and their corresponding collagen (blue in I) area statistics (J). The black pentagram represents the necrotic core. (K,L), Immunohistochemical results of MMP‐9 in aortic root (K) and corresponding positive area statistics (L). All data are presented as the mean ± SD (*n* = 3, 5, 6). ^*^ *p* < 0.05, ^**^ *p* < 0.01, ^***^ *p* < 0.001 and ^****^ *p* < 0.0001, and ns means no significance.

The therapeutic efficacy of nano‐Senotherapy on AS was also evaluated, encompassing plaque burden, necrotic core area, smooth muscle cell proliferation, collagen deposition, and plaque stability (Figure 9).

As shown in Figure 9A, ORO staining reflects the content of lipids (such as cholesteryl esters and triglycerides) within tissues. Lipid deposition in the aortas of AS mice indicates substantial plaque formation, with lesions appearing multifocal and scattered. According to Figure 9B, the lesion area in aortas of PBS‐treated AS mice reached nearly 39.48%. All four treatment groups showed significant differences compared to the PBS group, with values of 24.53%, 29.53%, 19.99%, and 8.23%, respectively. Notably, the nano‐Senotherapy formulations exhibited a synergistic effect, and the targeting capability of N@K_2_A@NEM conferred a distinct advantage. Consistent results were observed in ORO‐stained sections of the aortic root and their quantification (Figure 9C,D). Particularly noteworthy is the observation that after treatment with two nano‐Senotherapy at the 18th week, the lesion area in the aortic roots of AS mice were even smaller than that at the start of the intervention at the 13th week. This regression trend is more encouraging and exciting than merely slowing the progression of AS (Figure S18).

Meanwhile, the accumulation of substantial cholesterol crystals, calcium salts, or cellular debris within plaques contributes to the formation of a necrotic core. Hence, the necrotic core area serves as another important indicator for evaluating AS. The H&E staining of aortic root sections revealed numerous vacuolar structures—indicative of the necrotic core—within the vessels of PBS‐treated AS mice (Figure 9E, locations marked by black pentagram). Statistical analysis of the necrotic core area showed that nano‐Senotherapy also significantly reduced this parameter in AS mice. N@K_2_A@NEM performed most effectively, markedly decreasing the necrotic core area from 16.53 × 10^4^ µm^2^ in the PBS group to 6.67 × 10^4^ µm^2^, which can be attributed to its potent reduction of lipid deposition. Together, these findings demonstrated that nano‐Senotherapy can markedly reduce lipid peroxidation deposition and plaque formation in the aorta through synergistic mechanisms, and that NEM coating further enhances these therapeutic effects.

In addition, vascular smooth muscle cells (VSMC) are one of the major cellular components of the vascular wall. Their proliferation leads to wall thickening and lumen narrowing, an early hallmark of AS. Therefore, the extent of VSMC proliferation reflects AS progression, and α‐smooth muscle actin (α‐SMA) serves as a marker for VSMC proliferation. As shown in Figure 9G,H, the α‐SMA^+^ area in PBS‐treated AS mice increased nearly 6.88‐fold compared to the control group. All four treatment groups exhibited significant reductions to varying degrees. Among them, N@K_2_A@NEM again demonstrated the most pronounced effect, reducing the area by 64.05% and 31.18% relative to the PBS and N@K_2_A groups, respectively. Simultaneously, the accumulation of connective tissue components such as collagen fibers, synthesized by VSMC, is recognized as another step in AS development. In early stages, increased collagen contributes to fibrous cap formation. Figure 9I,J presents Masson‐stained sections of the aortic root and the corresponding quantification of collagen deposition. PBS‐treated AS mice showed nearly 9.52‐fold higher collagen deposition than normal mice. Compared to the PBS group and the two single‐agent treatment groups, nano‐Senotherapy substantially reduced collagen deposition. The N@K_2_A@NEM group decreased collagen to only 0.21‐fold that of the PBS group and, notably, only 51.38% of the level in the N@K_2_A group. These results indicate that nano‐Senotherapy significantly reduces fiber cap formation. We believe that, especially in the early stage of AS, this reflects the slow formation of plaque, which may not be a signal of the increased vulnerability of plaque [48].

Furthermore, the rupture of vulnerable plaques is a primary cause of other severe cardiovascular events. Therefore, reducing the formation of vulnerable plaques represents a key strategy in AS treatment. Among various factors, MMP‐9 serves as a critical marker for evaluating plaque vulnerability, as it significantly increases the risk of plaque rupture by degrading the fibrous cap. As shown in Figure 9K,L, the MMP‐9 positive area in the aortic roots of PBS‐treated AS mice increased 4.95‐fold compared with the control group, while all four treatment groups markedly reduced MMP‐9 expression. Although no significant difference was observed between the N@K_2_A and KYC groups, N@K_2_A@NEM—enhanced by biomimetic NEM coating for improved targeting—demonstrated a more pronounced reduction, with its MMP‐9 positive area only 1.63‐fold higher than that of the control group. These results indicate that nano‐Senotherapy effectively enhances plaque stability in AS mice, thereby lowering the risk of plaque rupture and preventing more serious clinical outcomes.

## 3 Conclusion

As atherosclerotic plaques progressively enlarge, they can narrow or even completely occlude major arteries in the body, such as the coronary, carotid, and renal arteries. The resulting severe CVDs are a leading cause of disability and mortality worldwide, making the prevention and treatment of AS critically important. Although current lipid‐lowering pharmacotherapy remains the mainstream approach for managing or controlling AS with the goal of inhibiting disease progression, it overlooks a key issue: the persistent presence of plaques—which cannot be resolved or reversed—may be a major reason for the limited long‐term benefits of these strategies. From a pathological perspective, the mechanisms driving the initiation and progression of AS can be broadly summarized as “continuous generation of foam cells driven by excess cholesterol, coupled with impaired clearance of these cells”. This indicates that foam cell formation and clearance represent central regulatory targets in AS. Therefore, we aimed to introduce a senotherapy strategy to mitigate or even reverse AS.

Utilizing the aromatic thioketal (ATK) unit previously developed in our laboratory, the MPO inhibitor KYC was engineered into a ROS‐responsive dimeric prodrug, K_2_A. Through intermolecular interactions—including hydrophobic forces, hydrogen bonding, and *π*–*π* stacking—K_2_A co‐assembles with Nav, a senolytic agent, to form nanoscale self‐assemblies designated N@K_2_A. Finally, a cell membrane coating technology was employed to generate N@K_2_A@NEM, a biomimetic nano‐Senotherapy capable of providing natural targeting to AS lesions and prolonging retention and accumulation at the diseased site. Impressively, both in vitro and in vivo experiments demonstrated that N@K_2_A@NEM exhibited superior efficacy in reducing senescence markers (SA‐β‐gal, p16, p21, and CD9) within aortic tissues. Consequently, N@K_2_A@NEM not only markedly attenuated lipid droplet‐induced foam cell formation, collagen deposition, and smooth muscle cell proliferation, but also reduced plaque vulnerability, thereby helping to prevent plaque rupture and subsequent severe CVDs. More importantly, the designed N@K_2_A@NEM mitigated the documented thrombocytopenia associated with Nav, offering a promising avenue for the clinical translation of Nav.

In addition, we have added a comprehensive comparison (Table S1) and an expanded discussion to benchmark our system against three categories of therapies: (I) conventional lipid‐lowering and anti‐inflammatory therapies, (II) previously reported nanotherapeutic strategies, and (III) representative senolytic approaches, including Nav and (Dasatinib + Quercetin).

Conventional therapies such as statins primarily reduce cholesterol levels, stabilize plaques, and slow disease progression, but typically exhibit limited regression capacity and require long‐term administration (months to years). Anti‐inflammatory therapy (e.g., canakinumab) has demonstrated modest clinical benefits, such as ∼15% reduction in CVDs, but does not directly eliminate senescent cells and is associated with increased infection risk. In contrast, our strategy is designed to address a key limitation of current therapies by directly targeting and eliminating senescent cells, which are increasingly recognized as critical drivers of AS progression. Compared with conventional approaches that mainly delay disease progression, this mechanism enables a more direct intervention at the level of disease pathology. Furthermore, compared with free Nav, our N@K_2_A@NEM system demonstrates significantly enhanced therapeutic efficacy (approximately 50% reduction in lesion area vs. ∼30% for the free drug), which we attribute to improved targeting and accumulation at diseased sites, as supported by the biodistribution and colocalization analyses. Importantly, the therapeutic response in our system is achieved within a relatively short treatment window (weeks), indicating a favorable turnaround time compared to conventional therapies that typically require long‐term administration.

Admittedly, several challenges remain for the clinical translation of N@K_2_A@NEM, including formulation stability during human application, cell membrane sourcing, design of the dosing regimen, regulatory approval status of the active pharmaceutical ingredient, and unknown side effects. Nevertheless, overall, this study not only presents an engineered, pathologically inspired biomimetic nano‐Senotherapy as a promising candidate for alleviating or even reversing AS, but also provides a valuable reference for research into other aging‐related diseases.

## 4 Experimental Section

For detailed experimental methods, please refer to the Supporting Information. All materials and instruments used are detailed in the Tables S2–S4. The number of samples for each experiment is detailed in Table S5.

## Author Contributions

Y.T., Y.Y., and Q.L. contributed to experiment conduct, data acquisition, analysis, and interpretation. Y.H. and Q.H. contributed to cell experiment conduct. S.R., S.Z., and X.Y. contributed to interpretation. S.L., and Q.Z. contributed to conception and design, drafted and revised the manuscript.

## Funding

This work was supported by National Natural Science Foundation of China [82204304, 82404874], Natural Science Foundation of Sichuan [2025ZNSFSC1733, 2026NSFSC0626], Sichuan Province Traditional Chinese Medicine Research Project [25MSZX428], Chengdu Medical Research Project [2024036], Dazhou Science and Technology Project [25YYJC0026], Dazhou Traditional Chinese Medicine Research Project [2025LHZRZD02], Youth Innovation Project of Sichuan Medical Association [Q22006], Scientific and Technological Innovation Team for Qinghai‐Tibetan Plateau Research in Southwest Minzu University [2024CXTD15], and Sichuan Province Youth Medical Science Research Project [20250717‐01].

## Ethics Statement

All animal experiments were approved by the Animal Care and Use Committee of Southwest Minzu University (2022MDLS17) and conducted in accordance with guidelines.

## Conflicts of Interest

The authors declare no conflicts of interest.

## Supporting information




**Supporting File**: advs75850‐sup‐0001‐SuppMat.docx.

## Data Availability

The data that support the findings of this study are available from the corresponding author upon reasonable request.
