# Supplementary material for: Pathological Mechanism‐Inspired Biomimetic Nano‐Senotherapy for Reversing Experimental Atherosclerosis in ApoE−/− Mice
Source: Adv Sci (Weinh). 2026 May 25:e75850. Online ahead of print. doi: 10.1002/advs.75850 (PMC13336057; doi:10.1002/advs.75850)
Supplement: Supplementary file 1 — Supporting File: advs75850‐sup‐0001‐SuppMat.docx. [file ADVS-9999-e75850-s001.docx]

**Supporting Information**

**Pathological Mechanism-Inspired Biomimetic Nano-Senotherapy for Reversing Experimental Atherosclerosis in ApoE^-/-^ Mice**

Yuhan Tian ^1^, Yanrui Yang ^2^, Qiuyu Li ^1^, Ying Hu ^1^, Qin Hu ^1^, Shasha Ran ^1^, Siyu Zhou ^1^, Xiujuan Yang ^2^, Shanshan Li ^1 *^, Qixiong Zhang ^2, 3, *^

1. College of Pharmacy and Food, Key Laboratory of Research and Application of Ethnic Medicine Processing and Preparation on the Qinghai-Tibet Plateau, Southwest Minzu University, Chengdu 610000, China
2. Department of Pharmacy, Personalized Drug Research and Therapy Key Laboratory of Sichuan Province, Sichuan Academy of Medical Sciences & Sichuan Provincial People’s Hospital, School of Medicine, University of Electronic Science and Technology of China, Chengdu 610000, China
3. Sichuan Provincial People’s Hospital East Sichuan Hospital & Dazhou First People’s Hospital, Dazhou 635000, China

^*^Corresponding authors:

Qixiong Zhang, PhD, Prof.,

Department of Pharmacy, Personalized Drug Research and Therapy Key Laboratory of Sichuan Province, Sichuan Academy of Medical Sciences & Sichuan Provincial People’s Hospital, School of Medicine, University of Electronic Science and Technology of China, Chengdu 610000, China

E-mail: qixiongzhang@outlook.com, zhangqixiong@uestc.edu.cn

Shanshan Li, PhD, Prof.,

College of Pharmacy and Food, Key Laboratory of Research and Application of Ethnic Medicine Processing and Preparation on the Qinghai-Tibet Plateau, Southwest Minzu University, Chengdu 610000, China

E-mail: sslscu2009@163.com

**Contents**

[Experimental Section 3](#_Toc227967268)

[Supplementary Figures 16](#_Toc227967269)

[Supplementary Tables 32](#_Toc227967270)

[References 35](#_Toc227967271)

# Experimental Section

*Materials*

The aromatic thioketal (ATK, refer as 4,4’-((9H-fluorene-9,9-diyl)bis(sulfanediyl))dibutyric acid) was prepared by our lab [1-4]. 3-(3-Dimethylaminopropyl)-1-ethylcarbodiimide hydrochloride (EDCI, Cat#105901) and *N*-hydroxysuccinimide (NHS, Cat#1094748) were purchased from Shanghai Adamas Reagent Co., Ltd. (China). Navitoclax (Nav, Cat#A276560) was obtained from Shanghai Aladdin Bio-Chem Technology Co., Ltd. (China). KYC (N-acetyl lysyltyrosylcysteine amide, Cat#175306) was purchased from GL Biochem (Shanghai) Ltd. (China). Phorbol 12-myristate 13-acetate (PMA, Cat#P8139) were purchased from Shanghai Titan Scientific Co., Ltd. (China). Polyethylene glycol 300 (PEG300, Cat#HY-Y0873) was obtained from MedChemExpress (USA). Cyanine7 NHS ester (Cy7-NHS, Cat#25020) and Cyanine5 NHS ester (Cy5-NHS, Cat#23020) were purchased from Lumiprobe Corporation (USA). All other reagents were of analytical grade, commercially available, and used as received unless otherwise specified.

Senescence β-Galactosidase Staining Kit (Cat#9860) was purchased from Cell Signaling Technology (USA). SuperKine Ultra-sensitive Cell Counting Kit-8 (CCK-8, Cat#BMU106-CN) was obtained from Abbkine, Inc. (USA). Oxidized low-density lipoprotein (ox-LDL, Cat#YB-002) and native low-density lipoprotein (LDL, Cat#YB-001) were obtained from Yiyuan Biotechnologies (China). Percoll (Cat#P8370) was obtained from Beijing Solarbio Science & Technology Co., Ltd. (China). Quantitative enzyme-linked immunosorbent assay (ELISA) kits for mouse cytokines and inflammatory markers, including IL-6 (Cat#EMC004.48), IL-1β (Cat#EMC001b.48), TGF-β1 (Cat#EMC107b.48), IL-8 (Cat#EMC104.48), and MPO (Cat#EMC045.48), were purchased from NeoBioscience Technology Co., Ltd. (China) and used according to the manufacturers’ instructions. Mouse Bcl-2 ELISA kit (Cat#CSB-E08855m) was purchased from Cusabio Inc. (China).

Ultimate pentafluorophenyl (PFP) column (5 μm, 4.6 × 250 mm, Cat#00224-31043) was purchased from Welch Materials, Inc. (USA). Polycarbonate track-etched membranes (19 mm diameter, pore size 0.1 μm, Cat#PCTE190100; 400 nm) were obtained from AVESTIN, Inc. (Canada). The RAW264.7 murine macrophage cell line (Cat#ZQ0098, RRID:CVCL_0493) was purchased from Zhongqiao Xinzhou Biotechnology Co., Ltd. (China). Apolipoprotein E knockout (ApoE^⁻/⁻^) mice were obtained from SpePharm (Beijing) Biotechnology Co., Ltd. (China; production license No. SPF20250618-01ZYH). The remaining reagents and instruments used are detailed in **Table S2-S4**.

*Synthesis and Characterization of Dimeric Prodrug (K_2_A)*

K_2_A was synthesized via an amide coupling reaction between ATK and KYC. ATK (M_w_ = 402.52 Da, 100 mg, 0.25 mmol) and 1-(3-dimethylaminopropyl)-3-ethylcarbodiimide hydrochloride (EDCI, M_w_ = 191.70 Da, 384.6 mg, 2.0 mmol) were accurately weighed and dissolved in a mixed solvent of N,N’-dimethylformamide (DMF, 6 mL) and deionized water (1 mL). The mixture was sonicated until a clear and homogeneous solution was obtained. The reaction solution was cooled in an ice–water bath (0 ℃) and stirred for 10 min to activate the carboxyl groups, followed by further activation at room temperature for 30 min. EDCI was employed as a mild carbodiimide coupling reagent to activate the carboxyl groups for subsequent amide bond formation. Subsequently, N-hydroxysuccinimide (NHS, M_w_ = 115.10 Da, 117.2 mg, 1.0 mmol) was added to the reaction mixture and stirred at room temperature for an additional 30 min to enhance the coupling efficiency. KYC (M_w_ = 453.49 Da, 274.0 mg, 0.60 mmol) was then added to the activated solution, and the reaction mixture was stirred at room temperature for 20 h. Upon completion, the reaction mixture was transferred into a dialysis bag (MWCO = 500 Da) and dialyzed against ultrapure water for 72 h to remove unreacted small molecules and by-products. The dialyzed solution was collected into cryovials, and subsequently lyophilized to afford the desired K_2_A dimeric prodrug as a pale solid (196.58 ± 7.23 mg (n = 3), yield of 62.15 ± 2.30%). The synthesis and chemical structure of K_2_A were confirmed by UV-vis, FT-IR, ^1^H-NMR, and ESI-MS analyses.

*Molecular Dynamics Simulations*

The initial structures for the molecular dynamics (MD) simulations in this study consisted of K_2_A and the ligand Nav. Initially, three independent replicate simulations were performed under neutral conditions. In addition, to accurately reflect the acidic environment established in the experiments, an additional simulation group was prepared where the protonation states of all molecules were strictly adjusted to pH 5.0. Due to its terminal modifications and side-chain cross-linking, K_2_A was assigned a neutral state with a net charge of zero at pH 5.0. For the ligand Nav, which contains multiple ionizable groups, the Dimorphite-DL algorithm on the PlayMolecule platform was utilized to predict its major microspecies at pH 5.0. The prediction indicated that the acylsulfonamide group of Nav underwent deprotonation, while the corresponding aliphatic secondary/tertiary amine groups were protonated, resulting in a stable zwitterionic structure. The generated 3D coordinates and partial charge assignments were saved in MOL2 format for subsequent parameterization.

Because both K_2_A and Nav contain highly modified, non-standard chemical structures, the General AMBER Force Field version 2 (GAFF2) was employed for their parameterization. The precise partial charges of the molecules were calculated using the AM1-BCC quantum chemical method. Force field topology and coordinate files were generated using the acpype package and subsequently converted into GROMACS-compatible formats.

The complex systems were assembled using the GROMACS 2025.4 software package. The AMBER ff14SB force field was applied as the overall simulation environment. The complexes were centered in a cubic simulation box with a minimum distance of 1.2 nm between the solute and the box edges. Three independent replicates were constructed by assigning different random seeds during the system initialization phase. The systems were explicitly solvated using the TIP3P water model. Subsequently, counterions (Na^+^ and Cl^-^) were added by replacing water molecules to neutralize the net charge of the systems and to mimic a physiological salt concentration of 0.15 M.

The solvated systems first underwent energy minimization using the steepest descent algorithm, with a maximum force threshold set to 1000 kJ/mol/nm to eliminate steric clashes and inappropriate atomic contacts. Subsequently, position-restrained equilibration simulations were performed. This included a 100 ps NVT (constant number of particles, volume, and temperature) equilibration to stabilize the system temperature at 298 K using the V-rescale thermostat, followed by a 100 ps NPT (constant number of particles, pressure, and temperature) equilibration to maintain the system pressure at 1 bar using the C-rescale barostat. Upon the removal of position restraints, a 300 ns production MD simulation was carried out for each system. Long-range electrostatic interactions were computed using the Particle Mesh Ewald (PME) method with a cutoff radius of 1.2 nm; the van der Waals force cutoff was correspondingly set to 1.2 nm. All covalent bond lengths were constrained using the LINCS algorithm, and the integration time step was set to 2 fs.

*Preparation and Characterization of nano-Senotherapy N@K_2_A*

The nanotherapeutic N@K_2_A was prepared by a nanoprecipitation–induced self-assembly method. Dimethyl sulfoxide (DMSO) was selected as the organic solvent to dissolve the K_2_A prodrug and the senolytic agent Navitoclax (Nav), as both compounds are insoluble in water. Ultrapure water was used as the antisolvent phase to induce polymer precipitation and nanoparticle formation. Briefly, 10 mL of ultrapure water was preheated to 50 ℃ and maintained under magnetic stirring. K_2_A (10 mg) and Nav (1 mg) were co-dissolved in DMSO to form the organic phase. The resulting solution was added dropwise into the aqueous phase at a flow rate of 0.1 mL min^-1^ under continuous stirring (120 rpm) at 50 ℃. The mixture was allowed to self-assemble for 10 min to form N@K_2_A nanoparticles. After self-assembly, the nanoparticle suspension was transferred into a dialysis bag (M_W_CO = 3500 Da) and dialyzed against deionized water overnight to remove free drug molecules and residual organic solvent. The purified N@K_2_A were obtained for further characterization.

The physicochemical properties of N@K_2_A were systematically characterized. The hydrodynamic diameter, polydispersity index (PDI), and zeta potential of N@K_2_A were measured at room temperature by Laser Particle Size Analyzer, measurements were performed at predetermined time points (0, 1, 2, 3, 4, and 5 days). The morphology was observed by TEM after negatively stained, and EDS mapping analysis of N@K_2_A element distribution.

*In Vitro ROS-Responsive Drug Release Study*

The ROS-responsive drug release behavior of N@K_2_A was evaluated using H_2_O_2_. Briefly, 5 mL of freshly prepared N@K_2_A suspension was loaded into dialysis bags (M_W_CO 3500 Da). The dialysis bags were immersed in 5 mL of PBS (pH 7.4) containing 1 mM H_2_O_2_ as the release medium. PBS (pH 7.4) without H_2_O_2_ was used as the control group. The dialysis systems were incubated at 37 ℃ with gentle shaking at 150 rpm. At predetermined time points (1, 2, 3, 4, 5, 6, 7, 8, 9, 10, 12, 18, 24, and 36 h), 200 μL of the release medium was withdrawn and replaced with an equal volume of fresh release medium to maintain sink conditions. The collected samples were mixed with 800 μL of HPLC-grade acetonitrile and analyzed by HPLC at a detection wavelength of 320 nm to determine the concentration of released Nav. The cumulative release percentage of Nav was calculated and plotted as a function of time.

Drug loading content (DLC), encapsulation efficiency (EE), and ROS-responsive drug release profiles were evaluated using established methods. All experiments were performed in triplicate.

$$Drug Loading Content \left( DLC \right)=\frac{m_{Nav,total}-m_{Nav,free}}{m_{nano-Senotherapy}}\times100\%$$

$$Encapsulation Efficiency \left( EE \right)=\frac{m_{Nav,total}-m_{Nav,free}}{m_{Nav,total}}\times100\%$$

*Cell Culture*

The murine macrophage cell line RAW264.7 was purchased from Zhongqiao Xinzhou Biotechnology Co., Ltd. (Shanghai, China). Cells were cultured in DMEM supplemented with 10% FBS, 100 U/mL penicillin, and 0.1 mg/mL streptomycin. RAW264.7 cells were maintained in a humidified incubator at 37 ℃ with 5% CO_2_ atmosphere, and the culture medium was replaced every 2-3 days. Cells in the logarithmic growth phase were used for all experiments.

*Cytotoxicity Assay*

The cytotoxicity of free Nav and N@K_2_A was evaluated using a Cell Counting Kit-8 (CCK-8) assay. RAW264.7 cells were seeded into 96-well plates at a density of 1 × 10⁴ cells per well and incubated overnight at 37 ℃ in a humidified atmosphere containing 5% CO₂ to allow cell attachment. After incubation, the culture medium was replaced with fresh medium containing free Nav or N@K_2_A at different concentrations (0-16 μM, calculated based on Nav equivalents). Cells were incubated with the formulations for 24 h. Untreated cells were used as the Control group. Subsequently, fresh culture medium containing CCK-8 reagent was added to each well at a volume ratio of 9:1 and incubated at 37 ℃ for 30 min. The absorbance was measured at 450 nm using a microplate reader. Cell viability was calculated as the percentage of absorbance relative to the control group.

$$Cell viability\left( \% \right)=\frac{A_{450(Sample)}-A_{450(Blank)}}{A_{450(Control)}-A_{450(Blank)}}\times100\%$$

*Cellular Uptake of* *nano-Senotherapy by Flow Cytometry*

To investigate the cellular uptake behavior of nano-Senotherapy, Cy5-labeled N@K_2_A was prepared. Briefly, Cy5 was co-dissolved with the components during nanoparticle preparation, followed by dialysis (MWCO 3500 Da) to remove free dye until no detectable fluorescence was observed in the dialysate. RAW264.7 macrophages were seeded in 6-well plates and allowed to adhere overnight under standard culture conditions. Cells were then incubated with Cy5@N@K_2_A at concentrations of 0.5, 1, 2, 4, and 8 μg/mL for 4 h at 37 ℃ in a humidified atmosphere containing 5% CO_2_. After incubation, cells were washed three times with ice-cold PBS to remove unbound nanoparticles. Cells were subsequently harvested, resuspended in PBS, and analyzed by flow cytometry. Cy5 fluorescence was detected using the appropriate red fluorescence channel, and nanoparticle uptake was quantified as the mean fluorescence intensity (MFI). Relative cellular uptake was expressed by normalizing the MFI of each group to that of the lowest concentration group. All experiments were performed independently in triplicate.

*In Vitro MPO-Mediated Foam Cell Senescence Model and Intervention*

RAW264.7 cells were seeded in 6-well plates at a density of 2 × 10⁵ cells per well and allowed to adhere for 24 h. To establish an MPO-mediated oxidative microenvironment, cells were pretreated with 100 nM PMA for 48 h to enhance endogenous MPO expression [5]. Native 1 mg/mL LDL was supplemented with 100 μM H_2_O_2_ and 100 mM NaCl as a chloride source. The LDL/H_2_O_2_/NaCl mixture was added directly to the cultured cells, allowing in situ MPO-mediated LDL oxidation within the cellular microenvironment [6-7]. After 24 h incubation with the LDL/H_2_O_2_/NaCl system, RAW264.7 macrophages exhibited pronounced lipid accumulation and senescence-associated features, indicating successful establishment of a senescent foam cell model (model group). Following model establishment, cells were divided into the following treatment groups: treated with 50 μg/mL free KYC or N@K_2_A (equivalent to free KYC) for 24 h.

The activity and content of MPO were measured using a commercial activity assay kit according to the manufacturer’s instructions. Briefly, cells were collected, lysed on ice, and centrifuged to obtain supernatants. The enzymatic activity was normalized to cell number and expressed as U/10⁶ cells.

Foam cell formation was evaluated by ORO staining to visualize intracellular lipid droplet accumulation. After the indicated treatments, cells were gently washed twice with PBS and fixed with ORO fixative for 20-30 min at room temperature. The fixative was then removed, and cells were rinsed twice with PBS to completely eliminate residual fixative. Subsequently, cells were briefly treated with 60% isopropanol for 20-30 s to facilitate lipid staining. After rapid removal of isopropanol, freshly prepared ORO working solution was added to each well and incubated for 10-20 min. Excess staining solution was discarded, and cells were washed with 60% isopropanol until the background became clear, followed by washing with ddH_2_O 2-3 times to remove residual dye. Finally, cells were covered with ddH_2_O and observed under optical microscope. Lipid accumulation was identified as red-stained intracellular lipid droplets. Quantitative analysis of ORO-positive area was performed using ImageJ software and expressed as the percentage of stained area relative to the total area.

Cellular senescence was assessed by senescence-associated β-galactosidase (SA-β-gal) staining following the indicated treatments. Briefly, cells were washed with PBS and fixed using SA-β-gal fixation solution for 15 min at room temperature. After fixation, cells were rinsed with PBS, and freshly prepared SA-β-gal staining working solution was added. Cells were then incubated at 37 ℃ overnight in a dry incubator protected from CO_2_. The SA-β-gal^+^ cells, indicated by blue staining, were visualized under optical microscope. Senescence levels were quantified as the percentage of SA-β-gal^+^ cells relative to the total number of cells.

To further evaluate the senescence-associated secretory phenotype (SASP), cell culture supernatants were collected after the indicated treatments, centrifuged at 3000 rpm for 10 min at 4 ℃ to remove cellular debris, and stored at -80 ℃ until analysis. The concentrations of SASP-related cytokines, including TGF-β1, IL-6, IL-8, and IL-1β, were quantified using enzyme-linked immunosorbent assay (ELISA) kits according to the manufacturers’ protocols.

*In Vitro ox-LDL-Induced Senescent Foam Cell Model and Intervention*

RAW264.7 cells were seeded in 6-well plates at a density of 2 × 10⁵ cells per well and allowed to adhere overnight. To synchronize cellular metabolic status, cells were subjected to serum starvation in DMEM for 10 h prior to foam cell induction. Subsequently, cells were incubated with 100 μg/mL ox-LDL for 24 h at 37 ℃ with 5% CO_2_, resulting in the formation of lipid-laden foam cells accompanied by cellular senescence [8-9]. After successful model establishment, cells were divided into the following treatment groups: treated with 1.0 μM free Nav or N@K_2_A (equivalent to free Nav) for 24 h.

After the indicated treatments, RAW264.7 cells were collected, washed with PBS, and stained with a fixable viability dye to exclude dead cells. Cells were then fixed and permeabilized, followed by intracellular staining with an anti-Bcl-2 antibody according to the manufacturer’s instructions. Flow cytometry was performed, and Bcl-2 expression was quantified as mean fluorescence intensity (MFI) in viable cells. Cellular senescence was assessed by SA-β-gal staining. For cell viability analysis, RAW264.7 cells were seeded in 96-well plates (1 × 10^4^ cells per well) and treated according to the same modeling and dosing protocol. After treatment, CCK-8 reagent was added to each well (10 μL/100 μL medium) and incubated at 37 ℃ for 1-2 h. Absorbance was measured at 450 nm using a microplate reader, and cell viability was calculated relative to untreated controls.

*Preparation and* *Characterization of NE and NEM*

Bone marrow-derived NE were isolated from ApoE^⁻/⁻^ mice as previously described, with minor modifications [10-12]. Briefly, mice were sacrificed by cervical dislocation and sterilized by immersion in 75% ethanol for 10-20 min. The femurs and tibias were carefully excised, washed sequentially with ethanol and phosphate-buffered saline (PBS), and transferred into RPMI 1640 medium. Both ends of the bones were cut to expose the marrow cavities, which were flushed repeatedly with fresh RPMI 1640 medium using a sterile 2 mL syringe until the bones appeared pale. The collected bone marrow suspension was passed through a 200-mesh cell strainer and centrifuged at 1000 rpm for 5 min to collect cells. The cell pellet was resuspended in PBS and washed twice by centrifugation at 1000 rpm for 3 mins. Finally, the cells were resuspended in 2 mL PBS to obtain a single-cell suspension. NE were further purified using a discontinuous Percoll density gradient centrifugation method.

A 100% Percoll stock solution was prepared by mixing Percoll with 10× PBS at a volume ratio of 9:1. The stock solution was further diluted with 0.15 M NaCl to prepare 78%, 65%, and 55% Percoll working solutions. Fifteen-milliliter centrifuge tubes were pre-rinsed with fetal bovine serum to minimize cell adhesion. Sequentially, 2 mL of 78% Percoll solution was added to the bottom of each tube, followed by careful layering of 2 mL of 65% and 55% Percoll solutions along the tube wall. Subsequently, 2 mL of the bone marrow cell suspension was gently layered onto the top of the 55% Percoll layer. Density gradient centrifugation was performed at 859 *g* for 30 min at room temperature without brake. Neutrophils were collected from the interface between the 78% and 65% Percoll layers as well as the upper region of the 78% layer. The collected cells were washed 3–4 times with PBS by centrifugation at 1000 rpm for 3 mins and resuspended in sterile water to obtain purified bone marrow–derived NE.

The purity of bone marrow-derived NE was assessed by flow cytometry. Briefly, freshly isolated cells were washed with PBS and resuspended in staining buffer. Cell suspensions were first gated based on forward and side scatter (FSC-A/SSC-A) to exclude debris, followed by singlet discrimination using FSC-H versus FSC-A. Cell viability was evaluated using Zombie NIR™ Fixable Viability Dye (BioLegend), and only live cells were included for subsequent analysis. Live cells were then stained with fluorochrome-conjugated antibodies against CD45, CD11b, and Ly6G. Leukocytes were identified as CD45^+^ cells, and neutrophils were defined as CD11b^+^Ly6G^+^ cells. Flow cytometric data were acquired using a flow cytometer and analyzed with FlowJo software. The purity of isolated NE was quantified as the percentage of CD11b^+^Ly6G^+^ cells among total live CD45^+^ cells.

NEM were extracted using a hypotonic lysis and differential centrifugation method [13-14]. A hypotonic lysis buffer (5 mL) was freshly prepared containing 225 mM D-mannitol, 75 mM sucrose, 30 mM Tris-HCl (pH 7.5), 0.2 mM EGTA, and a protease inhibitor cocktail. Freshly isolated neutrophils were washed 1-2 times with PBS and resuspended in the hypotonic lysis buffer. The cell suspension was homogenized using a Dounce homogenizer with a tight-fitting pestle for 40 strokes to disrupt the plasma membranes. The homogenate was centrifuged at 20,000 g for 30 min at 4 ℃ to remove intact cells and intracellular organelles. The supernatant was then further centrifuged at 120,000 g for 60 min at 4 ℃ to collect NEM. The membrane pellet was washed twice with PBS containing protease inhibitors and resuspended in PBS. To obtain NEM, the suspension was subjected to bath sonication for 3 min in sealed glass vials. The resulting NEM were stored at 4 ℃ for immediate use.

*Preparation and Characterization of Biomimetic nano-Senotherapy N@K_2_A@NEM*

Biomimetic nano-Senotherapy N@K_2_A@NEM was prepared by extrusion-based membrane coating. Briefly, freshly prepared N@K_2_A was mixed with NEM at a mass ratio of 1:1 in PBS (pH 7.4). The mixture was first sonicated in an ice-water bath for 2 min to promote membrane–nanoparticle fusion. Subsequently, the mixture was repeatedly extruded through a polycarbonate membrane (pore size ~400 nm) using a mini-extruder for 40 cycles. The resulting N@K_2_A@NEM was collected and stored at 4 ℃ in the dark for further characterization and biological experiments.

The total protein content of NEM and N@K_2_A@NEM was quantified using a BCA protein assay kit according to the manufacturer’s instructions. SDS-PAGE was performed to analyze the protein profiles of NEM, N@K_2_A@NEM. Equal amounts of protein were loaded onto polyacrylamide gels and separated under denaturing conditions. After electrophoresis, the gels were stained by Coomassie brilliant blue to visualize protein bands.

*In Vitro Hemocompatibility Assay*

The hemocompatibility of the nano-Senotherapy was evaluated by an in vitro hemolysis assay. Fresh whole blood was collected from male Sprague-Dawley (SD) rats (200-300 g) after anesthesia by intraperitoneal injection of 3% pentobarbital sodium. Blood was withdrawn from the abdominal aorta using a sterile 2.5 mL syringe and immediately transferred into sterile tubes without anticoagulant, followed by standing at room temperature for 4 h. The samples were centrifuged at 3000 rpm (approximately 1000 *g*) for 10 min to collect red blood cells (RBCs). The obtained RBCs were stored at 4 ℃ and used for subsequent hemolysis evaluation. Nanoparticle suspensions at different concentrations (10-200 μg/mL) were prepared in PBS. For each group, 1 mL of suspension was mixed with 20 μL of RBCs. Meanwhile, 1 mL of ddH_2_O and 1 mL of PBS mixed with 20 μL of RBCs were used as positive and negative controls, respectively. All samples were incubated at 37 ℃ for 4 h and then centrifuged at 3000 rpm for 15 min. The supernatants were carefully collected, photographed to visually record hemolysis, and transferred to a 96-well plate. The absorbance was measured at 542 nm using a microplate reader. The hemolysis percentage was calculated according to the following formula:

$$Hemolysis \left( \% \right)=\frac{A_{Sample}-A_{negative}}{A_{Positive}-A_{negative}}\times100\%$$

where $A_{\text{sample}}$, $A_{\text{negative}}$, and $A_{\text{positive}}$represent the absorbance of the nanoparticle-treated samples, PBS-treated samples, and ddH_2_O-treated samples, respectively.

*Short-Term in vivo Safety Evaluation*

To further evaluate the biosafety of the treatment regimen, a short-term safety study was conducted in healthy C57BL/6 mice following a dosing protocol consistent with the therapeutic study. Mice were randomly assigned to the indicated groups and received intravenous injections via the tail vein every 3 days (q3d schedule) on Days 1, 4, and 7. For the Nav, N@K_2_A, and N@K_2_A@NEM groups, formulations were administered at an equivalent dose of Nav (0.607 mg/kg), consistent with the dosing used in the therapeutic study. On Day 10, mice were anesthetized and blood samples were collected for hematological and biochemical analyses. Hematological evaluation included platelet count (PLT), white blood cell count (WBC), red blood cell count (RBC), mean platelet volume (MPV), platelet distribution width (PDW), and plateletcrit (PCT). Serum biochemical analysis included alanine aminotransferase (ALT), aspartate aminotransferase (AST), urea (UREA), and creatinine (CREA) to assess potential hepatic and renal toxicity. After blood collection, mice were euthanized, and liver and kidney tissues were harvested, fixed in 4% paraformaldehyde, embedded, sectioned, and subjected to hematoxylin and eosin (H&E) staining for histopathological evaluation.

*Establish the Mouse Model of AS*

Six-week-old male ApoE^⁻/⁻^ mice were used to establish the AS model. After one week of acclimatization, all mice were fed a high-fat diet (HFD; 1.25% cholesterol, 20% fat, 45% carbohydrate, and 23% protein; DC108C; from SpePharm (Beijing) Biotechnology Co., Ltd.) to induce atherosclerotic lesion formation. The HFD was continuously maintained throughout the modeling and treatment period.

*Ex Vivo Fluorescence Imaging for Biodistribution Evaluation*

To evaluate the plaque-targeting capability and biodistribution of the biomimetic nano-Senotherapy, N@K_2_A and N@K_2_A@NEM were fluorescently labeled with Cy7. Briefly, Cy7 was co-dissolved with Nav in DMSO during nanoparticle preparation. Excess free Cy7 was removed by dialysis (MWCO 3500 Da) until no detectable fluorescence was observed in the dialysate. Male ApoE^⁻/⁻^ mice were fed a HFD for 13 weeks to establish atherosclerotic lesions. Cy7@N@K_2_A or Cy7@N@K_2_A@NEM were administered via a single tail-vein injection at 2 μg Cy7. At predetermined time points post-injection (1, 3, 6, 9, and 12 h), mice were euthanized, and the entire aorta (from the aortic root to the iliac bifurcation) as well as major organs, including the heart, liver, spleen, lung, and kidney, were immediately harvested for ex vivo fluorescence imaging using an IVIS imaging system. Fluorescence signals were quantified as radiant efficiency within regions of interest (ROIs) using the imaging software. Time-dependent fluorescence accumulation curves and the area under the curve (AUC) were calculated to evaluate aortic targeting efficiency and organ biodistribution.

*In Vivo Treatment Protocol*

Male ApoE^⁻/⁻^ mice were fed a HFD to establish an AS model [15], and were randomly divided into six groups (n = 12): (i) Control, (ii) PBS, (iii) free Nav, (iv) KYC, (v) N@K_2_A, and (vi) N@K_2_A@NEM. After 13 weeks of HFD feeding, mice in all treatment groups received intravenous injections via the tail vein every 3 days for 6 consecutive weeks (from 13^th^ week to 18^th^ week). For the Nav, N@K_2_A, and N@K_2_A@NEM groups, formulations were administered at an equivalent dose of Nav of 0.607 mg/kg. The KYC group received KYC at a dose of 4.197 mg/kg, while the Control and PBS groups received no treatment or PBS injection, respectively. Body weight was recorded once per week throughout both the modeling and treatment periods to monitor animal health and treatment-related effects. At the conclusion of the treatment period, major organs, including the heart, liver, spleen, lungs, and kidneys, were dissected, weighed, and recorded. At the 18^th^ week, mice were euthanized, and tissues were collected for subsequent histological, biochemical, and imaging analyses.

*Animal allocation for endpoint analyses*

Sample size reporting was revised in accordance with ARRIVE 2.0 recommendations, and animal allocation was explicitly described for both whole-aorta and heart-based analyses [16].

A total of 12 mice were included in each group. A separate cohort of mice (n = 3 per group) was used for in vivo fluorescence imaging and was not included in the therapeutic cohort. After euthanasia, the whole aorta, the brachiocephalic artery, and the heart containing the aortic root were collected from each mouse and allocated to predefined downstream analyses. For whole-aorta analyses, separate subsets of aortas were used for en face Oil Red O staining (n = 3), en face SA-β-gal staining (n = 3), or ELISA analysis after liquid-nitrogen–ground whole aorta (n = 3). For aortic-root analyses, hearts were processed either for paraffin embedding followed by HE and immunohistochemical staining (n = 6) or for frozen embedding followed by Oil Red O and Masson staining (n = 6). In addition, frozen sections of the brachiocephalic artery were used for immunofluorescence analysis (n = 3). Therefore, the sample size based on aortic and cardiac detection reports reflects the predefined tissue allocation within the same study cohort, rather than individual animal cohorts inferred from the figure. Perfusion conditions were adjusted according to downstream applications, with PBS-only perfusion used for enzyme- and protein-sensitive assays (e.g., SA-β-gal and ELISA), and PBS followed by 4% PFA perfusion used for histological analyses. As shown in **Table S5**.

*Sample size determination*

Although a formal a priori power calculation was not documented before study initiation, the selected group size was based on prior literature, pilot observations, and common practice in atherosclerosis intervention/regression studies [17-18]. The primary endpoint was aortic root lesion area quantified by ORO staining. During study planning, a biologically meaningful difference of approximately 30–40%, with an expected variability of approximately 25–35% of the mean, was considered reasonable for this morphometric endpoint. Based on these assumptions, a group size of approximately 8–10 mice would generally be sufficient for the primary endpoint under a two-sided significance level of 0.05 and 80% power. To accommodate the complexity of the long-term HFD-fed in vivo study, repeated intravenous treatment, and predefined allocation of tissues to multiple downstream analyses, 12 mice were included in each group for therapeutic evaluation. A separate cohort was used for in vivo imaging.

*SA-β-gal Staining of Whole Aortas*

For SA-β-gal staining, mice were anesthetized and perfused with cold PBS for 5-10 min without paraformaldehyde (PFA) fixation. After perfusion, mice were immediately transferred to an ice-cold sterile surface, and the entire aorta was rapidly dissected under cold conditions. The isolated aortas were gently blotted dry, snap-frozen in liquid nitrogen (~15 s), and stored at −80 °C until use to preserve enzymatic activity. SA-β-gal staining was performed using a commercial staining kit following the manufacturer’s instructions. Briefly, tissues were fixed with SA-β-gal fixative at room temperature, washed with PBS, and incubated with freshly prepared staining solution (pH 6.0) at 37 ℃ overnight. Stained tissues were observed and imaged under an optical microscope.

*Immunohistochemical Analysis of Senescence-Related Markers*

For immunohistochemical analysis, mice were perfused with cold PBS followed by 4% PFA. Hearts containing the aortic root were harvested, post-fixed in 4% PFA, dehydrated, and embedded in paraffin. Serial sections of the aortic root were prepared for staining. Senescence- and inflammation-associated markers, including p21, p16, CD9, Ly6G, and MPO were detected using specific primary antibodies, followed by appropriate secondary antibodies. Signals were visualized using a DAB substrate, and images were captured using a microscope.

*Immunofluorescence Staining of Brachiocephalic Artery Sections*

To examine the distribution of senescent and apoptotic cells within atherosclerotic plaques, immunofluorescence staining was performed on frozen sections of the brachiocephalic artery. Briefly, brachiocephalic arteries were harvested after PBS perfusion, embedded in optimal cutting temperature (OCT) compound, and cryo-sectioned. Sections were fixed with 4% paraformaldehyde, washed with PBS, permeabilized with 0.1% Triton X-100, and blocked with 5% BSA or normal goat serum. The sections were then incubated overnight at 4 °C with a primary antibody against p16, followed by incubation with the corresponding fluorescence-conjugated secondary antibody. Apoptotic cells were detected using a TUNEL staining kit according to the manufacturer’s instructions. Nuclei were counterstained with DAPI. Representative images were acquired using a confocal laser scanning microscope under the same imaging settings for all groups.

*ELISA Analysis of Senescence-Associated Factors (Senescence-Associated Secretory Phenotype, SASP)*

For biochemical analysis, mice were perfused with cold PBS only, and whole aortas were rapidly excised on ice. After PBS washing, aortic tissues were cut into small fragments (10-20 mg), snap-frozen in liquid nitrogen or dry ice, and stored at -80 ℃. Tissue homogenates were prepared, and the levels of IL-6, IL-1β, IL-8, TGF-β1, MPO, and Bcl-2 were quantified using commercial ELISA kits according to the manufacturers’ protocols. All ELISA assays were completed within one week after sample collection to minimize protein degradation, with most samples analyzed within 3 days.

*ORO staining*

To assess atherosclerotic lesion burden, ORO staining of the whole aorta was performed. Mice were perfused with cold PBS for 10 min until complete removal of blood, followed by perfusion with 4% PFA for 20 min. Whole aortas were carefully dissected, cleaned of surrounding adipose tissue, fixed in 4% PFA, and stained with ORO to visualize lipid-rich lesions. In addition, ORO staining of aortic root cross-sections was performed on frozen sections prepared from hearts containing the aortic root.

*Histological and Immunohistochemical Assessment of Plaque Stability*

Plaque morphology and stability were further evaluated using hematoxylin and eosin (H&E) staining to assess necrotic core formation and Masson’s trichrome staining to evaluate collagen deposition. Immunohistochemical staining was performed to detect MMP-9 and α-smooth muscle actin (α-SMA) expression in aortic root sections, providing insight into plaque remodeling and inflammatory status.

*Quantification and Image Analysis*

Quantitative analyses of aortic staining, including ORO and SA-β-gal staining, as well as cross-sectional histological and immunohistochemical images (H&E, Masson’s trichrome, ORO, MMP-9, α-SMA, MPO, and CD9), were performed using ImageJ and Image-Pro Plus software. For en face staining and most cross-sectional analyses, lesion areas or positive staining areas were quantified as absolute areas and used for statistical comparison. In contrast, for nuclear senescence markers (p21 and p16), positive signals were quantified as the number of positively stained nuclei and expressed as the percentage of positive cells relative to the total number of nuclei within the lesion area.

*Statistical Analysis*

Variables with a normal distribution are presented as the mean ± SD. Two-tailed unpaired Student’s t test was used for comparisons of two groups, and one-way analysis of variance (ANOVA) was used for multiple comparisons. The p values less than 0.05 were considered statistically significant (*p < 0.05, **p < 0.01, ***p < 0.001, and ****p < 0.0001). All the statistical analyses were performed using GraphPad Prism 10.0 or Origin 2024 software.

# Supplementary Figures





**Figure S1**. Confirm the ROS-responsive hydrolysis process of K_2_A. **A**, ESI-MS spectrum of the hydrolysis products of K_2_A. **B**, Proposed chemical transformation process of K_2_A hydrolysis.


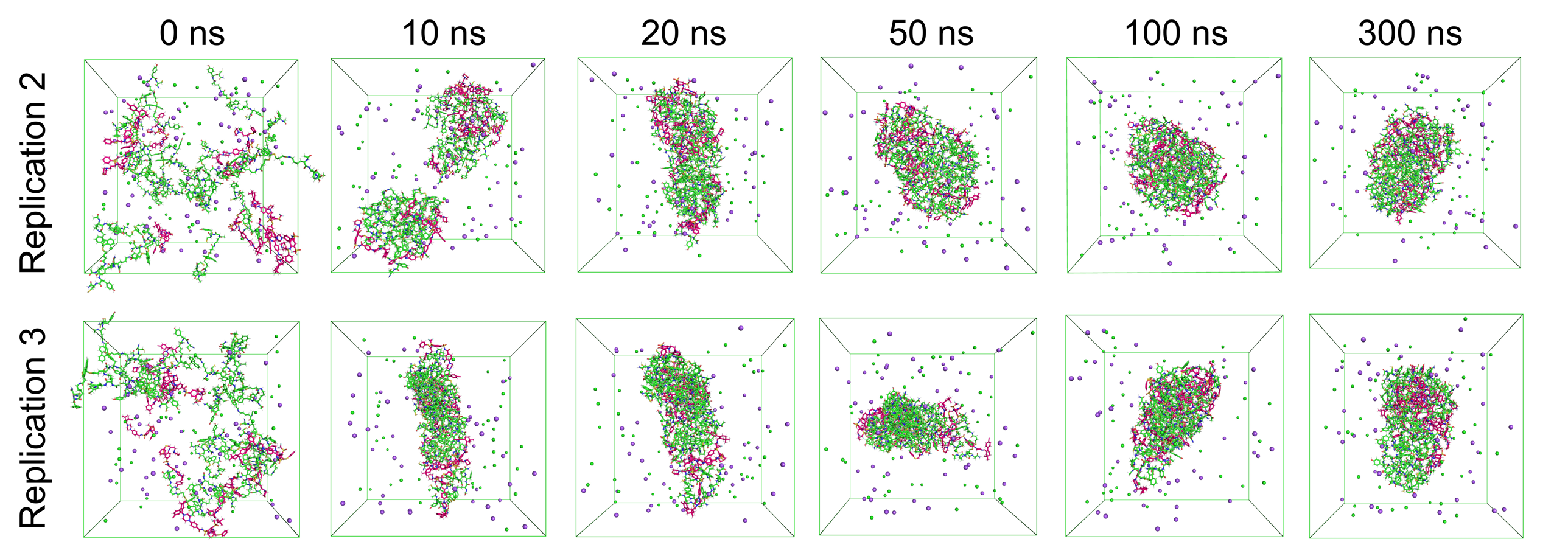


**Figure S2**. Molecular dynamics simulations of the self-assembled system composed of K_2_A and Nav.





**Figure S3**. **A**, Molecular dynamics simulation in pH 5.0 environment. **B**, Typical intermolecular interactions in the N@K_2_A structure include π-π stacking and hydrogen bonding. **C-G**, Changes in non-bonding interaction energy (C), Rg (D), SASA (E), and hydrogen bond (F-G) under pH 5.0 conditions.





**Figure S4**. EDS mapping images of the N@K_2_A.





**Figure S5**. **A-B**, The HPLC chromatograms of Nav (A) and the hydrolysis products of N@K_2_A (B). **C**, The standard curve of Nav.


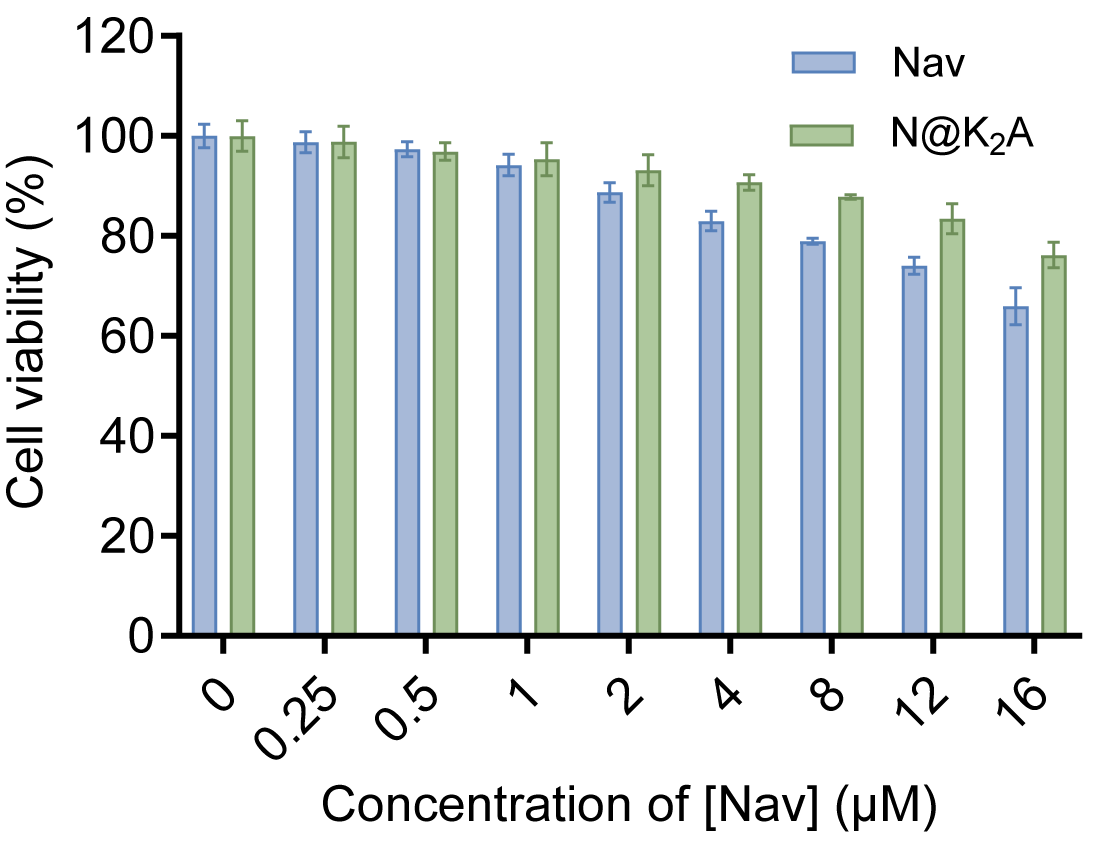


**Figure S6.** Cytotoxicity evaluation of two Nav formulations in RAW264.7 cells. Data are presented as mean ± SD (n = 6).


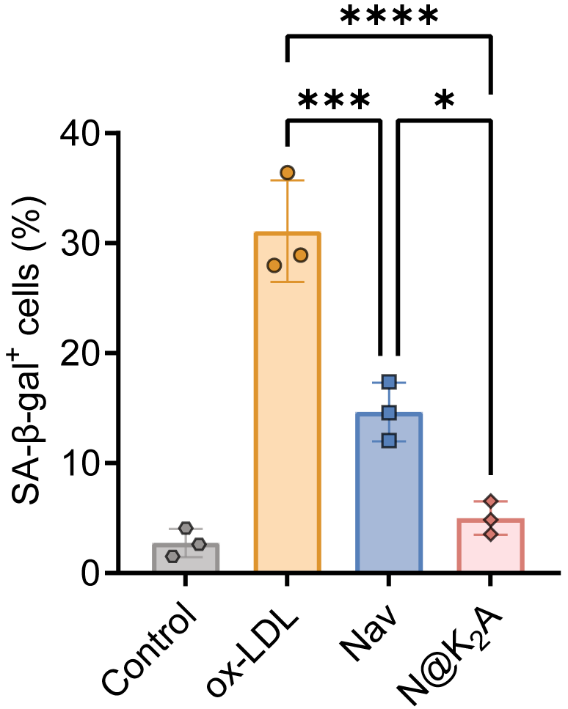


**Figure S7**. Quantitative analysis of the percentage of SA-β-gal^+^ RAW264.7 cells after ox-LDL stimulation and subsequent treatment with free Nav or N@K_2_A. Data are presented as mean ± SD (n = 3). *p < 0.05, ***p < 0.001, and ****p < 0.0001.

**
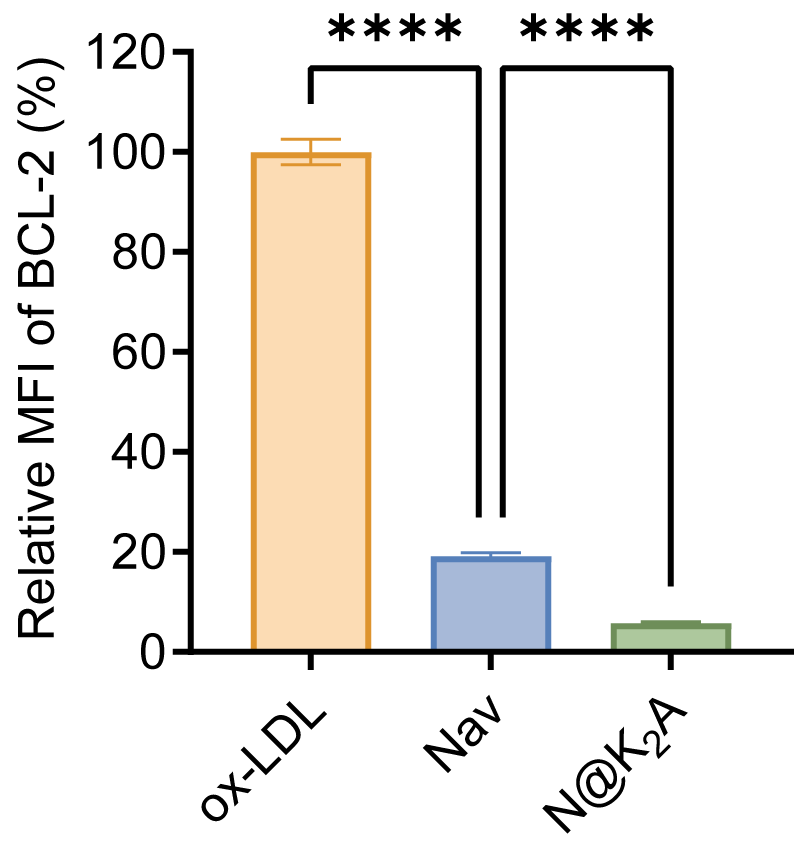
**

**Figure S8.** Flow cytometric analysis of Bcl-2 expression in ox-LDL–induced senescent RAW264.7 cells with different interventions. Quantitative analysis of relative MFI of Bcl-2 based on flow cytometry data. Data are presented as mean ± SD (n = 3). ****p < 0.0001.


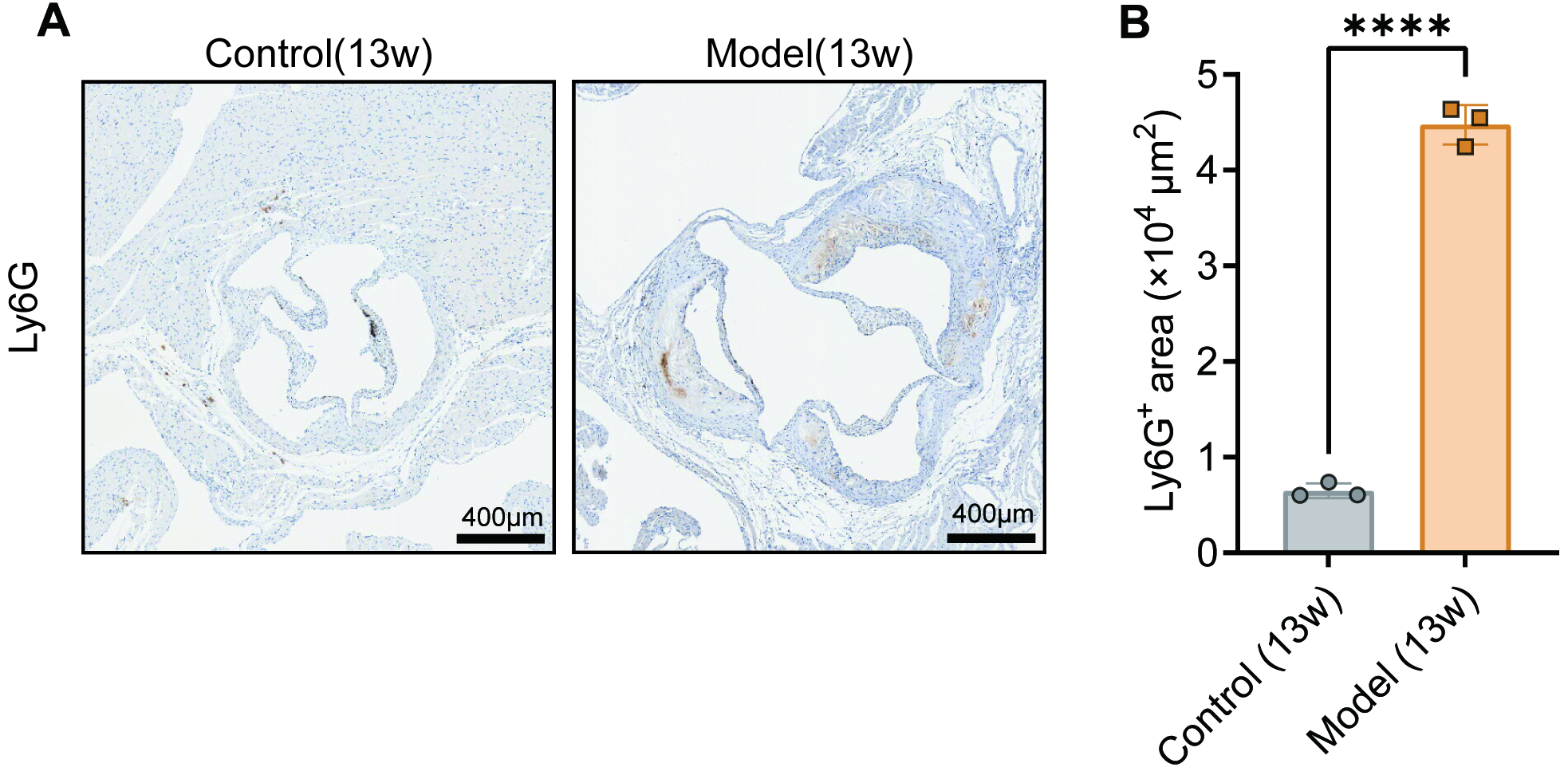


**Figure S9**. Representative immunohistochemical photographs (**A**) and positive area statistics (**B**) of Ly6G in aortic root. All data are presented the mean ± SD (n= 3). ****p < 0.0001.





**Figure S10.** Extraction of neutrophils (NE) and preparation of their cell membranes (NEM). **A**, Representative flow cytometry gating strategy for neutrophil identification. Cells were sequentially gated based on forward scatter (FSC) and side scatter (SSC) to exclude debris, followed by singlet discrimination and live-cell gating. CD45⁺ leukocytes were further analyzed for CD11b and Ly6G expression to identify neutrophils (CD11b⁺Ly6G⁺ population). **B**, Quantification of neutrophil purity expressed as the percentage of CD11b⁺Ly6G⁺ cells among total CD45⁺ cells. **C**, Representative bright-field optical microscopy image showing isolated NE after purification. Typical neutrophil morphology is indicated by arrows. Scale bar, 20 μm. **D**, Representative TEM image of neutrophil membrane vesicles (NEM) dispersed in PBS, exhibiting vesicular morphology with a hollow structure. Scale bar, 100 nm. Data are presented as mean ± SD (n = 3).


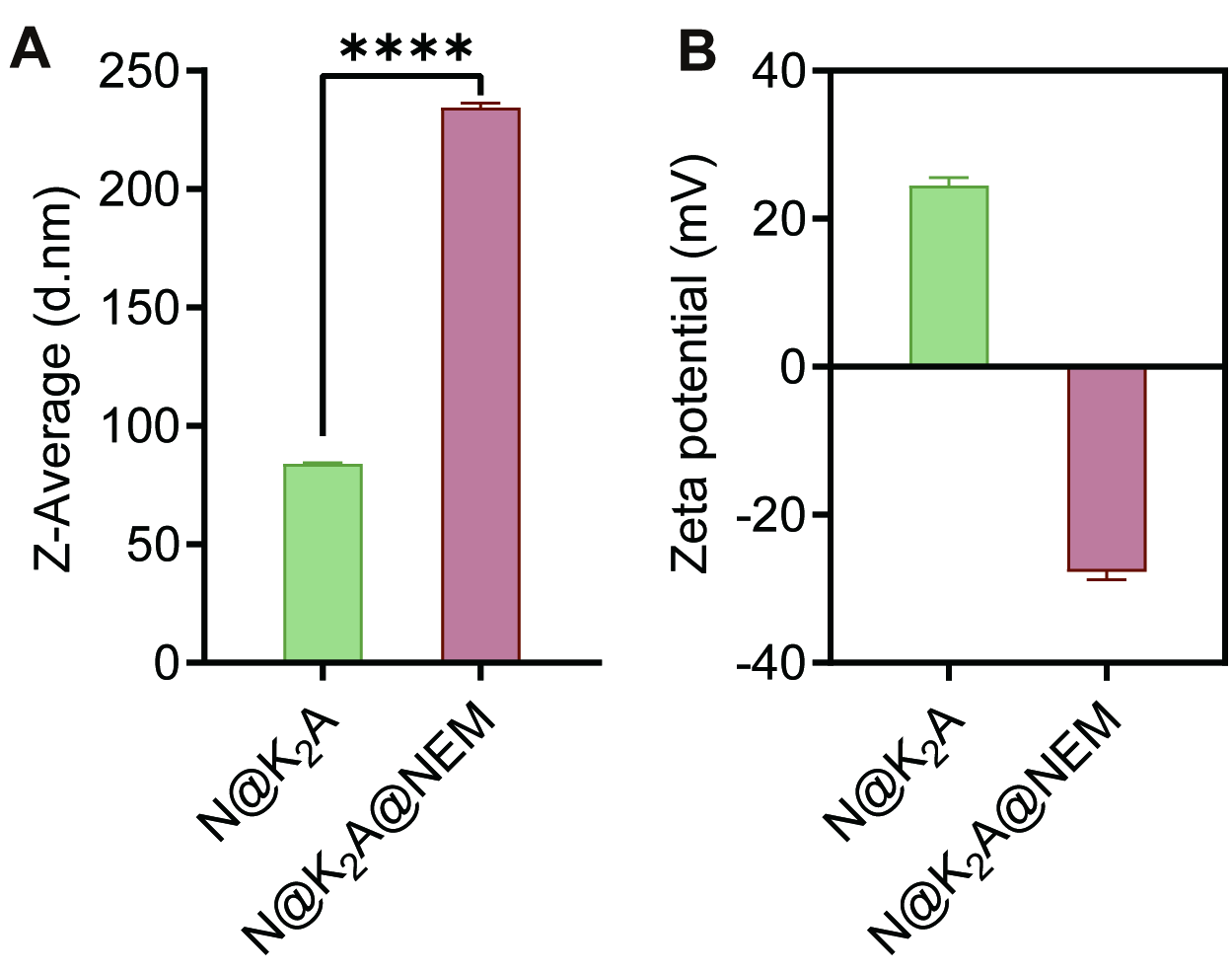


**Figure S11**. Comparison of nano-Senotherapy before and after covering NEM. The Z-average hydrodynamic diameter (**A**) and Zeta potential (**B**) of N@K_2_A and N@K_2_A@NEM measured by DLS. Data are presented as mean ± SD (n = 3). ****p < 0.0001.





**Figure S12.** In vitro hemolysis evaluation of N@K_2_A, and N@K_2_A@NEM. **A-B**, Representative photographs of red blood cells incubated with N@K_2_A, (A) and N@K_2_A@NEM (B) at indicated concentrations in PBS. ddH_2_O and PBS were used as positive and negative controls, respectively. **C,** Quantitative analysis of hemolysis induced by N@K_2_A and N@K_2_A@NEM at different concentrations. Hemolysis was calculated based on the absorbance of released hemoglobin and expressed as percentage. Data are presented as mean ± SD (n = 3).


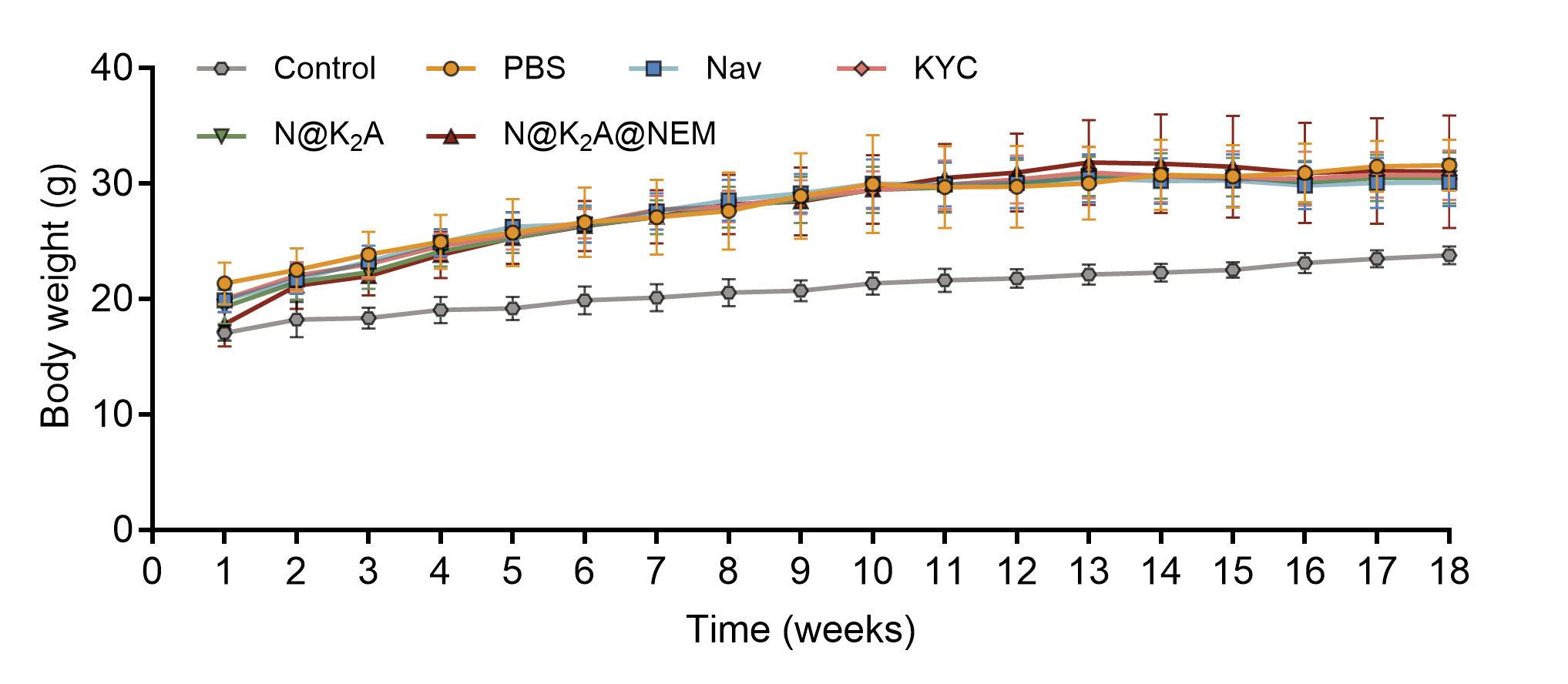


**Figure S13.** Body weight changes of ApoE^⁻/⁻^ mice during the experiment. Data are presented as mean ± SD (n = 12).


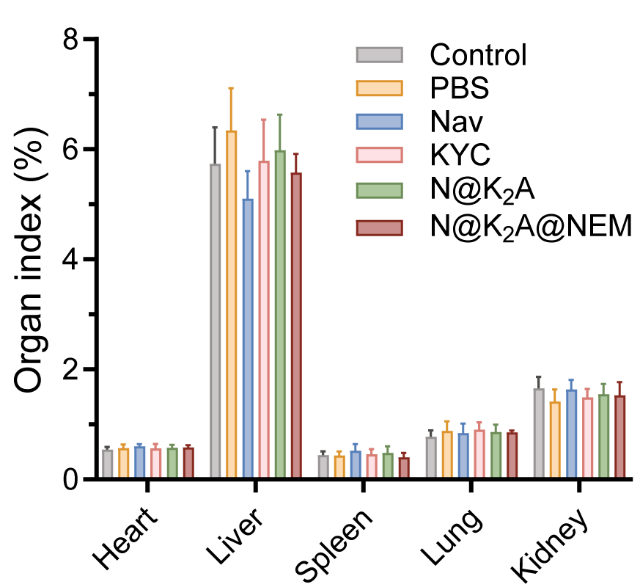


**Figure S14.** Organ index of major tissues after different treatments in ApoE^⁻/⁻^ mice. Organ index of major organs, including heart, liver, spleen, lung, and kidney, in AS mice following treatment with PBS, free Nav, KYC, N@K_2_A, or N@K_2_A@NEM. The organ index was calculated as organ weight normalized to body weight. Data are presented as mean ± SD (n = 6).





**Figure S15**. In vivo biosafety evaluation of different treatments. **A**, Schematic illustration of the short-term in vivo biosafety evaluation under the therapeutic dosing regimen. Healthy C57BL/6 mice received *i.v.* injections of 0.607 mg/kg Nav formulations on days 1, 4, and 7 (q3d), and blood and tissue samples were collected on day 10. **B**, Hematological and serum biochemical analyses were performed to assess systemic toxicity after repeated intravenous administration. Blood parameters included white blood cell count (WBC), red blood cell count (RBC), platelet count (PLT), mean platelet volume (MPV), platelet distribution width (PDW), and plateletcrit (PCT). Serum biochemical indicators included alanine aminotransferase (ALT), aspartate aminotransferase (AST), creatinine (CREA), and urea (UREA). **C-D**, Representative H&E staining images of liver (C) and kidney (D) tissues. Data are presented as mean ± SD (n = 5), ns means no significance.

**
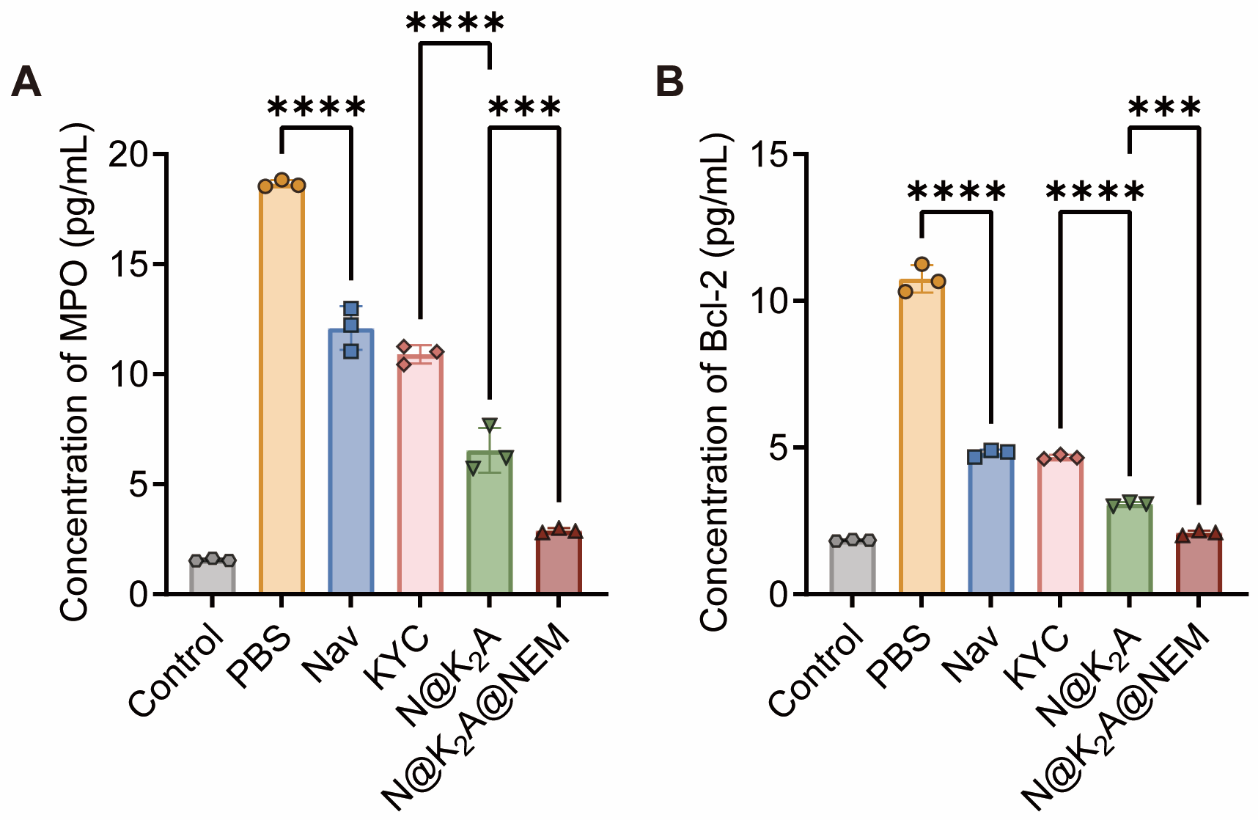
**

**Figure S16.** Concentration of MPO (**A**) and Bcl-2 (**B**) measured by ELISA in tissue homogenate suspensions collected from mice after different treatments. Data are presented as mean ± SD (n = 3). ***p < 0.001 and ****p < 0.0001.





**Figure S17**. The changes of SASP in the whole aorta tissue of mice treated with different formulations, including TGF-β1, IL-6, IL-8, and IL-1β. All data are presented the mean ± SD (n= 3). ^*^p < 0.05, ^**^p < 0.01, ^***^p < 0.001, ^****^p < 0.0001, and ns means no significance.


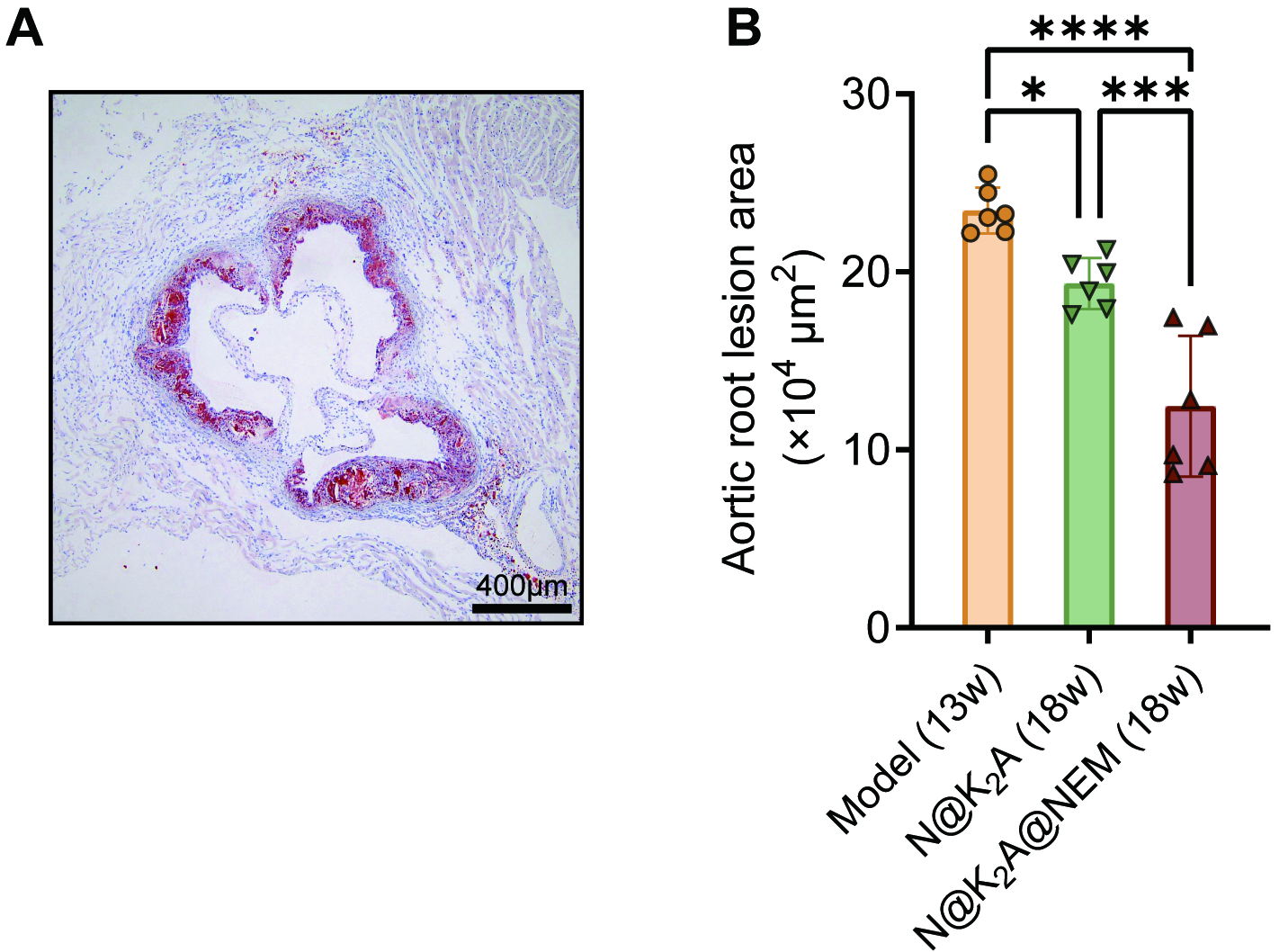


**Figure S18**. The representative photograph of ORO staining of aortic root tissue sections at 13^th^ week (**A**) and comparison of lesion area with that of the N@K_2_A or N@K_2_A@NEM treatment group at 18^th^ week (**B**). Data are presented as mean ± SD (n = 6). ****p < 0.0001.

# Supplementary Tables

**Table S1**. Comparison of conventional, senolytic, and nanomedicine-based therapies for AS.

| **Strategy** | **Representative therapy** | **Mechanism** | **Representative therapeutic benefit** | **Key limitations** | **Turnaround time** | **Ref.** |
| --- | --- | --- | --- | --- | --- | --- |
| Lipid-lowering | Statins | Cholesterol reduction | Moderate (10-30%) | No direct effect on senescent cells | Long-term (months to years) | 1. [JAMA. 2006;295(13):1556-1565](https://jamanetwork.com/journals/jama/fullarticle/202629); 2. [JAMA. 2007;297(5):499-508](https://jamanetwork.com/journals/jama/fullarticle/205424). |
| Anti-inflammatory | Canakinumab | IL-1β inhibition | Modest (~15% reduction in cardiovascular events) | Infection risk, no Senolytics | Long-term | 1. [New England Journal of Medicine. 2017;377(12):1119-1131](https://www.nejm.org/doi/full/10.1056/NEJMoa1707914); |
| Senotherapy | Dasatinib + Quercetin | Multi-pathway apoptosis | Variable (~20–30%) | Low specificity; off-target effects | Short-term  (days to weeks) | 1. [Aging Cell. 2016/10/01 2016;15(5):973-977.](https://onlinelibrary.wiley.com/doi/full/10.1111/acel.12458) |
| Senotherapy | Navitoclax | BCL-2/BCL-xL inhibition | Moderate (~20–30%) | Thrombocytopenia; poor targeting | Short-term  (days to weeks) | 1. [Aging Cell. 2015/08/01 2015;14(4):644-658.](https://onlinelibrary.wiley.com/doi/10.1111/acel.12344) 2. [Science 2016, 354, 472–477.](https://www.science.org/doi/10.1126/science.aaf6659) |
| Nanotherapy | Responsive nanocarriers | ROS scavenging + inflammatory modulation | Moderate (~20–40%) | No direct senescent cell clearance | Weeks | 1. [Nature Communications. 2014/01/20 2014;5(1):3065.](https://www.nature.com/articles/ncomms4065) |
| Nano-senotherapy | Senolytics + Senomorphics | Senotherapy + targeted delivery | High (~50% lesion reduction in aortic root) | To be further evaluated | Short-term (weeks) | This work |

**Table S2**. Reagents and materials for histological staining, protein analysis, dialysis, and biochemical assays.

| **Item** | **Company** | **Cat#** |
| --- | --- | --- |
| Oil Red O Stain Kit, For Cultured Cells | Beijing Solarbio Science & Technology Co., Ltd. (China) | G1262 |
| Masson’s Trichrome Stain Kit |  | G1340 |
| MPO Activity Assay Kit |  | BC5715 |
| Oil Red O Saturated Solution, 0.5% |  | G1260 |
| Standard Dialysis Tubing (MWCO 3,500), 25 mm | Shanghai Yuanye Bio-Technology Co., Ltd. (China) | MD3525-5m |
| Standard Dialysis Tubing (MWCO 8,000–14,000), 25 mm |  | MD1425-5m |
| Cellulose Dialysis Tubing (MWCO 100–500 Da), 24 mm |  | SP131057-0.5m |
| Coomassie Blue Fast Staining and No-decoloring Solution | Epizyme Biotech Co., Ltd. (China) | PS111 |

**Table S3**. Antibodies and related reagents.

| **Item** | **Company** | **Cat#** |
| --- | --- | --- |
| FITC Anti-Mouse CD45 Antibody [30-F11] | Elabscience Biotechnology Co., Ltd. (USA) | E-AB-F1136C |
| PE Anti-Mouse/Human CD11b Antibody [M1/70] |  | E-AB-F1081D |
| APC Anti-Mouse Ly6G Antibody [1A8] |  | E-AB-F1108E |
| FITC anti-Bcl-2 Antibody | Biolegend, Inc. (USA) | 633503 |
| Zombie NIR Fixable Viability Kit |  | 423105 |
| FOXP3 Fix/Perm Buffer Set |  | 421403 |
| Anti-p21 Antibody | Abclonal Technology Co., Ltd. (China) | A19094 |
| Anti-p16 Antibody |  | A11651 |
| Anti-CD9 Antibody | Servicebio Technology Co., Ltd. (China) | GB155697 |
| Anti-Ly6G Antibody |  | GB12229 |
| Anti-MMP9 Antibody | Proteintech Group, Inc. (China) | 27306-1-AP |
| Anti-α-SMA Antibody | Boster Biological Technology Co., Ltd. (China) | BM0002 |
| Anti-MPO Antibody | Abcam Limited. (UK) | ab208670 |

**Table S4**. Experimental instruments.

| **Instrument Name** | **Model** | **Company** |
| --- | --- | --- |
| Flow Cytometer | FACS Canto2 | Becton, Dickinson and Company (USA) |
| Multifunctional Full-Wavelength Microplate Reader | Cytation5 | BioTek Instruments, Inc. (USA) |
| Chemiluminescent Imaging System | Chemi Doc | Bio-Rad Laboratories, Inc. (USA) |
| Transmission electron microscope | JEM-2100Plus | JEOL (Japan) |
| Inverted Fluorescence Microscope | IX73 | Olympus Corporation (Japan) |
| Upright Fluorescence Microscope | BX53 | Olympus Corporation (Japan) |
| Stereo Microscope | SZX16 | Olympus Corporation (Japan) |
| High-Performance Liquid Chromatography | 1260 Infinity2 | Agilent Technologies, Inc. (USA) |
| Laser Particle Size Analyzer | Zetasizer Nano ZS | Malvern Panalytical Ltd. (UK) |

**Table S5**. The numbers shown are assay-specific sample sizes per group. Most ex vivo assays were performed on different tissues collected from the same study (n = 12 per group). In vivo imaging was conducted in live animals (parallel experiment) (n = 3 per group).

| **Category** | **Sample** **size (n)** | **Tissue/source** | **Notes** |
| --- | --- | --- | --- |
| In vivo imaging | 3 | Live animals (parallel experiment) | Live-animal imaging before sacrifice |
| En face ORO | 3 | Whole aorta | Whole-mount aortic staining |
| En face SA-β-gal | 3 | Whole aorta | Whole-mount aortic staining |
| ELISA | 3 | Liquid-nitrogen-ground aortic tissue | Aortic tissue homogenate |
| Paraffin HE + IHC | 6 | Paraffin-embedded aortic root sections from the heart | Section-based histology |
| Frozen ORO + Masson | 6 | Frozen aortic root sections from the heart | Section-based histology |
| Frozen IF | 3 | Frozen sections of the brachiocephalic artery | Artery branch dissected from the aorta |

# References

[1] Q. Zhang, S. Li, J. Ren, X. He, H. Shi, F. Zhang, H. Li, R. Tong, *J Control Release* **2022**, *348*, 22, <https://doi.org/10.1016/j.jconrel.2022.04.026>.

[2] Q. Zhang, Y. Tian, Y. Yang, Q. Huang, H. Feng, R. Zeng, S. Li, *J Nanobiotechnology* **2024**, *22* (1), 475, <https://doi.org/10.1186/s12951-024-02745-5>.

[3] S. Li, A. Xie, H. Li, X. Zou, Q. Zhang, *J Control Release* **2019**, *316*, 66, <https://doi.org/10.1016/j.jconrel.2019.10.054>.

[4] Q. Zhang, S. Li, L. Bai, D. Yu, H. Li, R. Tong, *ACS Appl Mater Interfaces* **2022**, *14* (1), 297, <https://doi.org/10.1021/acsami.1c20031>.

[5] H. L. Damascena, W. A. A. Silveira, M. S. Castro, W. Fontes, *Cells* **2022**, *11* (18), <https://doi.org/10.3390/cells11182889>.

[6] M. R. Rodrigues, D. Rodriguez, M. Russo, A. Campa, *Biochem Biophys Res Commun* **2002**, *292* (4), 869, <https://doi.org/10.1006/bbrc.2002.6724>.

[7] P. Van Antwerpen, K. Z. Boudjeltia, S. Babar, I. Legssyer, P. Moreau, N. Moguilevsky, M. Vanhaeverbeek, J. Ducobu, J. Neve, *Biochem Biophys Res Commun* **2005**, *337* (1), 82, <https://doi.org/10.1016/j.bbrc.2005.09.013>.

[8] X. Liang, J. Zhang, J. Yu, J. Zhao, S. Yang, *Eur J Med Res* **2025**, *30* (1), 359, <https://doi.org/10.1186/s40001-025-02562-y>.

[9] H. Cao, Q. Jia, L. Yan, C. Chen, S. Xing, D. Shen, *Int J Mol Sci* **2019**, *20* (23), <https://doi.org/10.3390/ijms20236093>.

[10] V. Kremer, O. Godon, P. Bruhns, F. Jonsson, L. de Chaisemartin, *Front Immunol* **2023**, *14*, 1301183, <https://doi.org/10.3389/fimmu.2023.1301183>.

[11] N. Zhang, T. Zhang, J. Feng, J. Shang, B. Zhang, Q. Dong, Z. Zhang, C. Sun, *Mater Today Bio* **2025**, *30*, 101397, <https://doi.org/10.1016/j.mtbio.2024.101397>.

[12] J. Wu, T. Ma, M. Zhu, J. Mu, T. Huang, D. Xu, N. Lin, J. Gao, *ACS Nano* **2024**, <https://doi.org/10.1021/acsnano.3c12694>.

[13] W. Wang, Y. Gao, M. Zhang, Y. Li, B. Z. Tang, *ACS Nano* **2023**, *17* (8), 7394, <https://doi.org/10.1021/acsnano.2c11762>.

[14] X. Dong, J. Gao, C. Y. Zhang, C. Hayworth, M. Frank, Z. Wang, *ACS Nano* **2019**, *13* (2), 1272, <https://doi.org/10.1021/acsnano.8b06572>.

[15] J. Zeng, Y. Zhang, Y. Gao, M. Jia, Y. Guo, X. Li, Y. Wang, C. Zhao, J. Qiu, S. McGinty, W. Miao, G. Wang, Y. Wang, *ACS Nano* **2025**, *19* (25), 22968, <https://doi.org/10.1021/acsnano.5c02492>.

[16] A. M. Garrido, A. Kaistha, A. K. Uryga, S. Oc, K. Foote, A. Shah, A. Finigan, N. Figg, L. Dobnikar, H. Jørgensen, M. Bennett, *Cardiovascular Research* **2022**, *118* (7), 1713, <https://doi.org/10.1093/cvr/cvab208>.

[17] M. Sharma, M. P. Schlegel, M. S. Afonso, E. J. Brown, K. Rahman, A. Weinstock, B. E. Sansbury, E. M. Corr, C. van Solingen, G. J. Koelwyn, L. C. Shanley, L. Beckett, D. Peled, J. J. Lafaille, M. Spite, P. n. Loke, E. A. Fisher, K. J. Moore, *Circulation Research* **2020**, *127* (3), 335, <https://doi.org/doi:10.1161/CIRCRESAHA.119.316461>.

[18] Z. He, W. Chen, K. Hu, Y. Luo, W. Zeng, X. He, T. Li, J. Ouyang, Y. Li, L. Xie, Y. Zhang, Q. Xu, S. Yang, M. Guo, W. Zou, Y. Li, L. Huang, L. Chen, X. Zhang, Q. Saiding, R. Wang, M.-R. Zhang, N. Kong, T. Xie, X. Song, W. Tao, *Nature Nanotechnology* **2024**, *19* (9), 1386, <https://doi.org/10.1038/s41565-024-01687-1>.
